# Observation versus self-perception: Effectiveness of measures to increase separate bio-waste collection from multi-storey residential buildings

**DOI:** 10.1177/0734242X261419233

**Published:** 2026-02-20

**Authors:** Konstantin Bachmann, Malek Simon Grimm, Ralf Wagner, David Laner

**Affiliations:** 1 Research Group on Resource Management and Solid Waste Engineering, Faculty of Civil and Environmental Engineering, University of Kassel, Kassel, Germany; 2 Chair of Sustainable Marketing, Faculty of Economics and Management, University of Kassel, Kassel, Germany

**Keywords:** Organic waste, source separation, impurities, behaviour intervention, characterization campaigns, structured interviews

## Abstract

Schemes for source separation of municipal bio-waste aim for maximum coverage and minimum contamination to enable high-quality recovery routes. To identify suitable approaches to improve the separate collection and quality of bio-waste in multi-storey residential buildings, which often exhibit poor source separation, this study evaluates the effectiveness of a set of measures aiming to sensitize residents and facilitate source separation at household level. Three buildings (181 residents) were selected in Kassel (Germany) using a purposive sampling approach. The measures implemented comprised verbal, written and visual communication as well as pre-sorting equipment. By conducting 8 waste characterization campaigns and 2 series of structured interviews (24 and 16 participants, respectively), the qualities and quantities of collected bio-waste were contrasted with the residents’ self-perceived level of information and participation in the source separation of bio-waste. Starting at 55%, 46% and 42%, separate collection rates for bio-waste increased to averages of 64%, 60% and 48%, respectively, after implementation of the measures. Impurity contents of separately collected bio-wastes were barely affected, averaging 2%, 8% and 4%, while peaking at 4%, 16% and 8%, respectively. Considering interview response patterns, the withdrawal of access to bio-waste bins from residents not interested in complying with source-separation guidelines seems necessary to achieve low contaminant levels. The residents’ perception of individual measures varied across the buildings, indicating a need for site-specific interventions to motivate people to participate in source separation. Further observations suggest that an intensified and sustained feeling of social responsibility and monitoring positively affects bio-waste separation behaviour.

## Introduction

Bio-waste accounts for over 50% of globally generated municipal solid waste (United Nations Environment Programme, 2024). If all bio-waste was separately collected and purposefully treated, an estimated amount of 643 million Mg could be diverted from landfills, mitigating greenhouse gas emissions related to global bio-waste management by −78% to −130% (Maletz et al., 2025). To reduce environmental harm and at the same time, promote a circular economy, the European Union mandated its member states to establish a separate bio-waste collection by 31 December 2023 (EU, 2008). Schemes for effective separate collection of bio-waste aim for maximum coverage regarding the generated bio-waste and minimum contamination of the separately collected bio-waste with impurities (cf. Trémier et al., 2025). Bio-waste collected together with other municipal solid waste flows cannot be specifically recovered and can itself hinder the recycling process (cf. Albizzati et al., 2024; Blasenbauer et al., 2024). The reduction of foreign materials in separately collected bio-waste is considered essential for producing high-quality compost because sorting and conditioning options in bio-waste treatment plants are technically and economically limited with respect to the reduction of impurity contents (cf. Alessi et al., 2020; Friege and Eger, 2022). In Germany, this is reflected in the national Bio-Waste Ordinance (BioAbfV; Bundestag, 2022), which stipulates thresholds for impurity contents of treatment products as well as treatment input materials in order to obtain an appropriate quality of products derived from bio-waste to be used on land. For household bio-waste, regulations specify a maximum total plastics content (>20 mm) of 1% and a maximum total impurities content of 3%, both at delivery. Otherwise, pre-treatment is necessary.

For good-quality municipal bio-waste, rigorous source separation at the household level is key. Therefore, factors influencing people’s waste separation behaviour have been studied comprehensively. In an anthropological analysis, Pedersen and Manhice (2020) reported that 7% of residents are very committed to waste separation, while 88% separate only convenient waste, and 5% do not separate at all. Barriers such as cultural perceptions, hygiene concerns, lack of convenience, and system mistrust were highlighted, suggesting that policies should focus on awareness, convenient routines, perceptions of order, and trust. Stoeva and Alriksson (2017) emphasized the importance of proper conditions for waste separation, noting that satisfaction with local conditions aligns behaviour with individuals’ attitudes towards recycling. Bernstad (2014) found written information ineffective at increasing food waste separation, stressing that convenience at household level is crucial. Rousta et al. (2017) underscored the necessity of convenient infrastructure, noting that reduced distances to collection points increase sorted waste volumes. Their review highlighted the importance of engaging users through personal contact and two-sided communication over economic incentives, while identifying socio-demographic and social pressure factors as context-dependent influences on sorting behaviour (Rousta et al., 2017). Consequently, being a result of people’s waste separation behaviour, bio-waste quantity and quality are affected by a variety of factors, including hygiene, convenience, knowledge, attitude towards waste management, socio-demographic aspects and social pressure.

Despite past efforts to enhance the separate collection of bio-waste in Germany (cf. LAGA, 2022), waste characterization campaigns regularly show that the source separation of bio-waste is often particularly poor in multi-storey residential buildings (MSRBs; cf. Sabrowski, 2020; Siepenkothen and Neumann, 2017; Siepenkothen et al., 2022). This is also the case for Kassel, a mid-sized German city with a population of ca. 200,000 inhabitants in which approximately one third of residential buildings are MSRBs (Stadt Kassel, 2022). Separate collection rates (SCRs) for bio-waste were found to be as low as 24% in large MSRBs (>10 households) with, at the same time, high levels of impurities (5–8%; Bachmann et al., 2025). Therefore, under consideration of the aforementioned factors affecting people’s bio-waste separation behaviour, it is the goal of this study to identify suitable approaches for improving the quality and SCR of bio-waste in MSRBs based on an evaluation of the effectiveness of a set of measures implemented at three sites in Kassel. The analysis builds on eight waste characterization campaigns regarding the quality and amounts of generated bio-waste as well as two series of structured interviews with residents on their perceived level of information and participation in the source separation of bio-waste between September 2023 and January 2025. While previous studies investigating bio-waste separation behaviour in MSRBs so far addressed either observed or perceived behaviour, this study investigates both observed and perceived bio-waste separation behaviour to contrast them with each other.

## Materials and methods

### Measures to foster the separate collection of bio-waste in MSRBs

**Figure 1.**
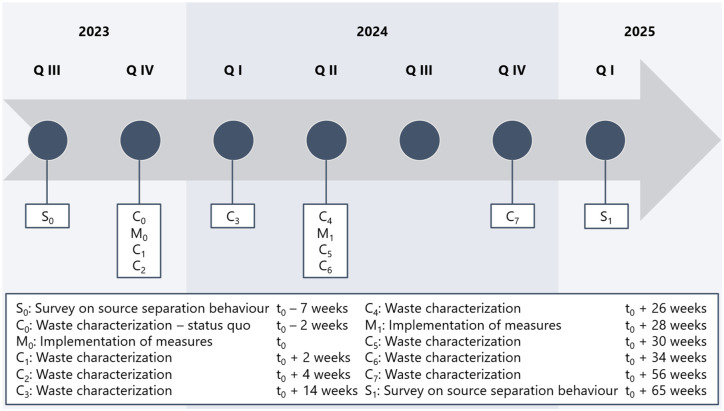
Timeline of implementation of measures (M), characterization campaigns (C) and surveys (S).

In collaboration with Kassel’s municipal waste management authority and in line with literature (cf. Bernstad, 2014; Pedersen and Manhice, 2020; Rousta et al., 2017; Stoeva and Alriksson, 2017), possible reasons identified for the relatively poor source separation in large residential buildings are the inconvenience of carrying bio-waste to collection points of the building complex, increased anonymity due to shared waste bins, demotivation caused by the apparent misbehaviour of other residents, as well as lack of knowledge or confusion about the local waste collection scheme. Correspondingly, a set of measures addressing these issues to enhance the source separation behaviour was designed and implemented (cf. Supplemental Material for pictures of the measures). The measures comprised door-to-door waste consultations of households, highlighting the general importance of separately collecting bio-waste with low levels of impurities. In addition, an official multilingual information leaflet on correct waste separation was handed out. Each household was given a pre-sorting bin with a capacity of 8 litres and 10 fitting paper collection bags for free to facilitate source separation at household level. To raise awareness about the benefits associated with separate bio-waste collection and recycling, posters were put up in the hallway close to the entrance of each building: a first poster with a question about nutrient recycling and energy recovery when the measures were implemented, and a second poster informing about the respective positive effects of separately collecting bio-waste 2 weeks later. Halfway through the investigation period (M1, cf. Figure 1), five more paper collection bags were distributed to each household, accompanied by a postal reminder on the importance of collecting bio-waste separately.

The above-described measures were implemented at three MSRBs in different parts of the city (cf. Supplemental Material regarding their location in Kassel). The selection criteria for the buildings comprised a similar number of households (>10), bio-waste bins provided at the building level (exclusive use), and locations in different parts of the city, while taking logistics into consideration so as to allow for waste collection at all sites simultaneously. Accordingly, three buildings with identical numbers of households and varying waste bin capacities (as provided by the municipal waste authority) were selected (cf. Table 1).

**Table 1.** Characteristics of the selected buildings.

Building	Storeys	Households	Residents	Total bin volume (litres)	
				Bio-waste	Residual waste
A	8	28	79	720	2200
B	4	28	62	600	2200/3300^a^
C	4	28	40	480	1100

a As of April 2024, the original container volume of 2200 litres was increased to 3300 litres.

### Waste characterization campaigns

To monitor the effectiveness of the measures regarding their suitability to foster separate bio-waste collection, eight waste characterization campaigns (C0–C7) were carried out over a period of 58 weeks (October 2023–November 2024) to assess waste qualities and amounts over time, starting by determining the status quo (C0) shortly before the initial implementation of measures (M0, cf. Figure 1). As bio-waste bins and residual waste bins are the major collection pathways for bio-waste collected at kerbside in Kassel (Bachmann et al., 2025), both separately collected bio-waste and residual waste were characterized to determine a potential shift of waste fractions between the two household waste flows. Because of organisational framework conditions, bio-wastes and residual wastes of all three buildings were collected on the same day, entailing that the waste had been collected for different durations since the last regular pick-up by the municipal waste collection service owing to varied official pick-up dates from site to site (cf. Supplemental Material for exact intervals). To account for this and the different numbers of residents, total amounts of waste were defined as the weights collected per capita per week. To assess the quality of separately collected bio-wastes in terms of impurities and connect them to thresholds stipulated by the German Bio-Waste Ordinance (Bundestag, 2022), fractional compositions (mass-based) were calculated.

In total, a provided bin volume of 62.8 m^3^ (bio-waste: 14.4 m^3^, residual waste: 48.4 m^3^) was sorted manually. The waste was collected by exchanging filled bins with empty bins and sorted in compliance with Germany-wide acknowledged guidelines on the characterization of municipal solid waste (cf. Bundesgütegemeinschaft Kompost eV, 2018; Sächsisches Landesamt für Umwelt, Landwirtschaft und Geologie, 2016). Prior to bio-waste sorting, collection bags contained in the bio-waste bins were sorted out, categorized by material (paper, bio-degradable plastic, non-degradable plastic), weighed to monitor the amounts of bio-waste collected via different types of bags and afterwards returned for sorting. The collected waste (separately for each bin) was screened at 40 mm (using a rotary drum screen) and 10 mm (using a sieving table), resulting in a coarse (>40 mm), a medium (10–40 mm) and a fine fraction (<10 mm). The coarse fraction was sorted into 18 categories for bio-waste and into 41 categories for residual waste. For the medium fraction, the composition was determined via a sample of 5–10 litres, which was sorted into 18 categories in the case of bio-waste and 14 categories in the case of residual waste. Weights were determined for all sorting categories. The fine fraction was collected, weighed, and discarded without further sorting. An overview on the sorting categories and the detailed results for each sorting campaign are provided as part of the Supplemental Material. Besides gardening waste as well as kitchen and other organic waste, also paper collection bags (recommended for bio-waste collection and suitable for composting) and particles <10 mm (mostly soil, lawn cuttings, fly larvae) are regarded as target materials in bio-waste bins. Due to the scope of the study, the analysis of residual waste characterizations is limited to the contained bio-waste.

### Door-to-door surveys on bio-waste separation behaviour

To investigate the residents’ perception of their own bio-waste separation behaviour, two household surveys were conducted at the three sites in September 2023 (prior to the first waste characterization campaign) and in January 2025 (after completion of all waste characterization campaigns). The surveys were designed as structured interviews with both closed-ended (single-choice) questions and open-ended questions. While the basic set of questions was the same in both surveys and addressed the residents’ knowledge, attitude and practices regarding the source separation of household (bio-)waste, the second survey additionally included questions on the perceived effectiveness of the measures implemented. All questions and responses can be found in the Supplemental Material. Responses to closed-ended questions were evaluated quantitatively by descriptive statistics, whereas responses to open-ended questions were evaluated qualitatively. During both surveys, each building unit was visited multiple times (on different days and at different times) to reach as many participants as possible. Responses obtained during the first survey (24 participating households) are used to evaluate the residents’ self-perceived bio-waste separation behaviour, while responses obtained during the second survey (16 participating households) are used to evaluate the residents’ own impressions concerning the impact of the measures implemented. Regarding socio-demographic aspects, responses from both surveys are used. As eight households participated in both surveys, in these cases, only responses from survey 1 are considered to avoid double-counting (cf. Table 2). Inferential statistical tests and significance tests between the two surveys are refrained from as the minimum sample size was not met (Schönbrodt and Perugini, 2013; Stewart et al., 2012).

**Table 2.** Number of participants and coverage of households (in brackets) during surveys.

Evaluated aspects	Surveyed households			
	Building A	Building B	Building C	Total
Self-perceived bio-waste separation behaviour (survey 1)	10 (36%)	6 (21%)	8 (29%)	24 (29%)
Self-perceived impact of implemented measures (survey 2)^a^	6 (21%)	4 (14%)	5 (18%)	15 (18%)
Socio-demographic aspects (surveys 1 and 2)^b^	12 (43%)	9 (32%)	11 (39%)	32 (38%)

a One of six households from building C did not state their opinion on the impacts of the implemented measures.

b Eight households participated in both surveys. In these cases, only responses from survey 1 are considered to avoid double-counting.

## Results

### Observed bio-waste separation behaviour

#### Separate collection rates of bio-waste

**Figure 2.**
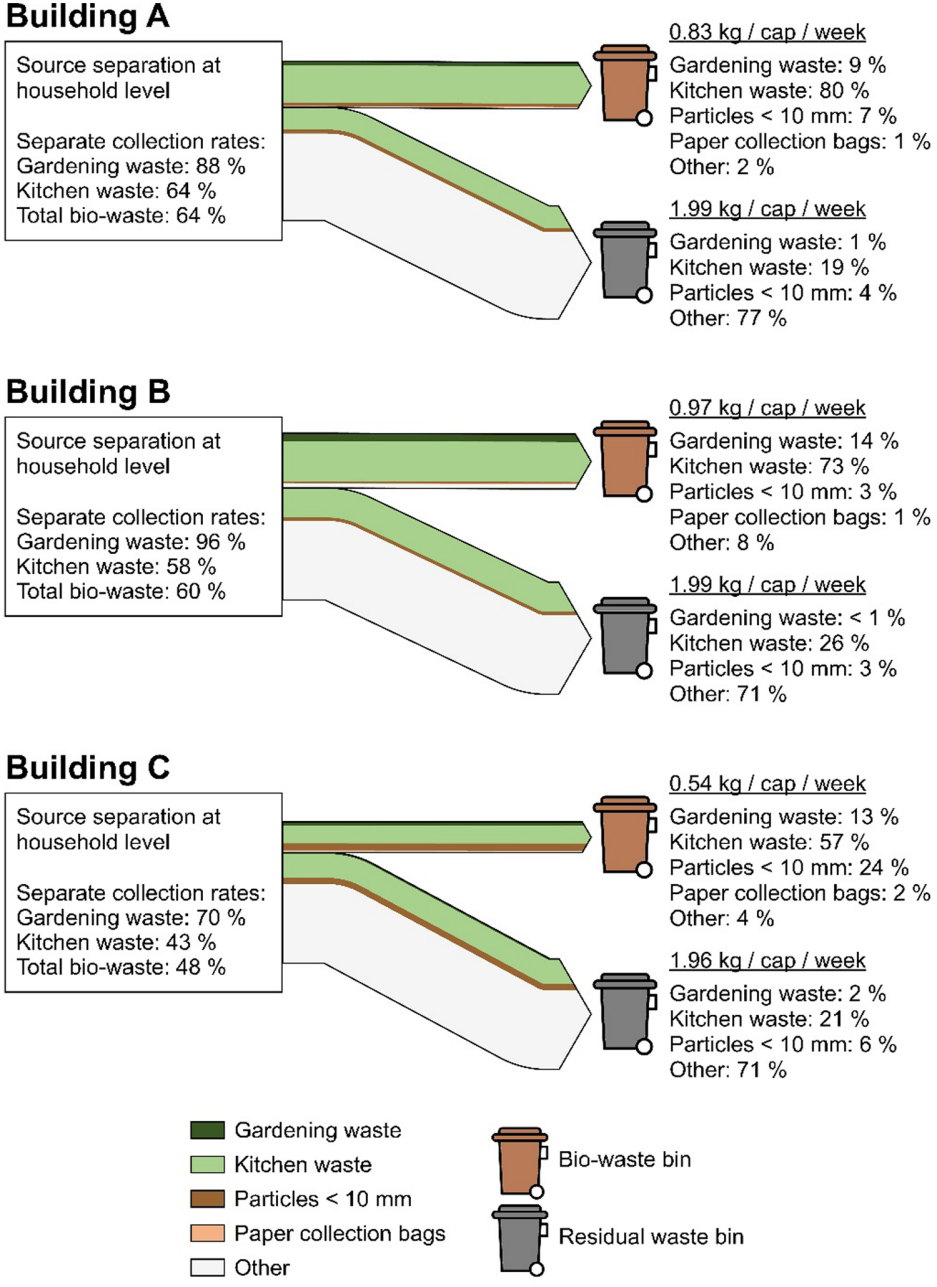
Average distribution of wastes to bio-waste bins and residual waste bins across all eight waste characterization campaigns. Campaigns C5 and C6 for building B are not considered because of irregular bio-waste collection prior to the campaigns. Campaign C7 for building C is not considered because of irregular residual waste collection prior to the campaign.

**Figure 3.**
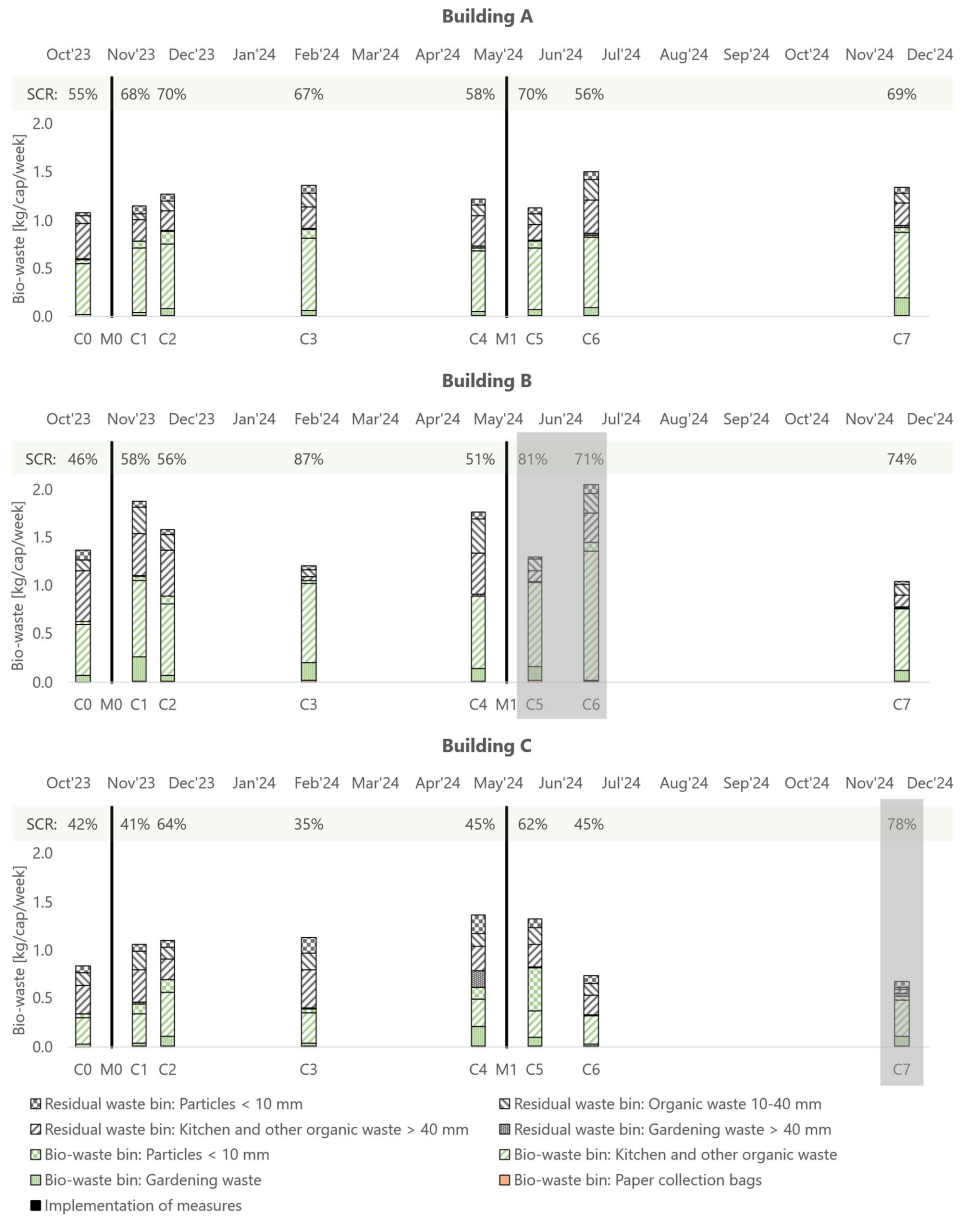
Collection of bio-waste via bio-waste bins and residual waste bins. Shaded areas display characterization results to be considered with caution because of irregular waste collection prior to the campaigns (building B, C5 and C6: irregular bio-waste collection; building C, C7: irregular residual waste collection). C0–C7: waste characterization campaigns; M0, M1: implementation of measures; SCR: separate collection rate.

In consideration of bio-waste bins and residual waste bins as the two major collection pathways for bio-waste, average SCRs across the eight waste characterization campaigns are 64%, 60% and 48% for buildings A, B and C, respectively (cf. Figure 2). After the implementation of measures, the separate collection of bio-waste increased in all buildings both times (cf. Figure 3, M0 and M1). In terms of mean SCRs determined during characterization campaigns C1 and C2, the first implementation of measures (M0, comprising door-to-door waste consultations, information leaflets, pre-sorting bins, paper collection bags and the first posters) led to an increase in separate collection by 24–25% compared to C0 in all three buildings. The second set of measures (M1, comprising paper collection bags, postal letters and the second posters) again increased the separate collection of bio-waste, but to varying extents. When comparing campaign C4 (prior to M1) to C5 and C6 (after M1), mean increases of 9% and 19% could be achieved for buildings A and C, respectively. For building B, an increase of 49% was determined. However, around the time of characterization campaigns C5–C6, the bio-waste bins of building B were emptied infrequently by the municipal waste collection service because impurity contents were determined to be unacceptably high. During this time, bio-waste bins showed comparatively high fill levels, and the provided volume of residual waste bins was increased by 1100 litres (+50%). These changes certainly affected the distribution of bio-waste to bio-waste bins and residual waste bins, limiting the validity of data related to C5 and C6 at building B.

#### Separately collected amounts of bio-waste

**Figure 4.**
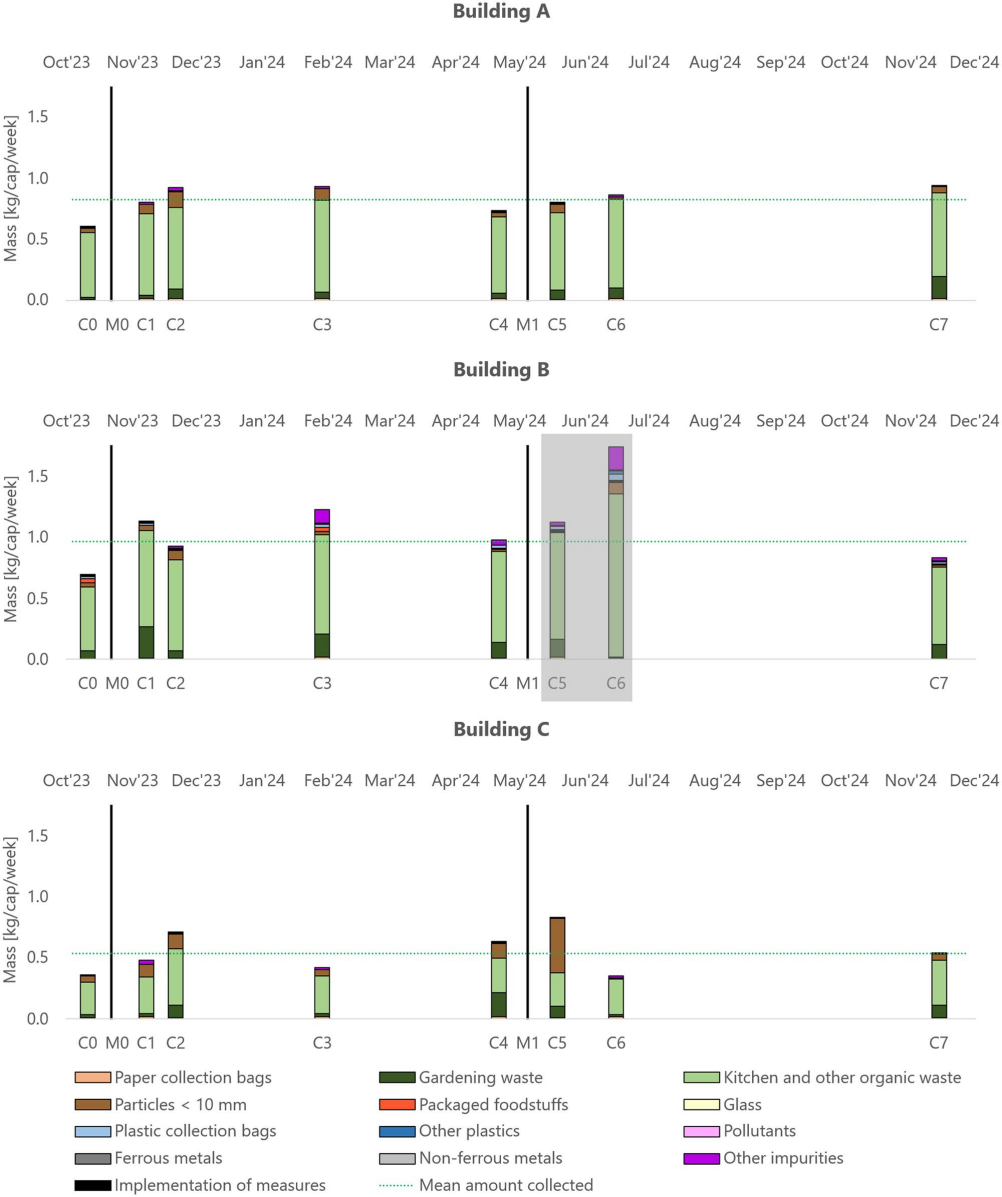
Amounts of separately collected bio-waste per capita per week. Results of C5 and C6 for building B (shaded area) are not considered in the calculation of the mean amount collected because of irregular bio-waste collection prior to the campaigns. C0–C7: waste characterization campaigns; M0, M1: implementation of measures.

At the beginning of the investigation period (C0), 0.61 and 0.70 kg of bio-waste (including impurities) were collected separately per capita per week in buildings A and B, respectively, and mean collection amounts of 0.83 and 0.97 kg cap^−1^ week^−1^ can be determined taking the entire investigation period into consideration (and not taking results of C5 and C6 for building B into account due to irregular bio-waste collection during that time, cf. Figure 4). In building C, substantially lower amounts of bio-waste were collected separately, starting from 0.36 kg cap^−1^ week^−1^ and providing a mean amount of 0.54 kg cap^−1^ week^−1^ across the entire investigation period. While the sum of gardening waste and fine particles (mostly soil, lawn cuttings, fly larvae) is similar across the three buildings (0.13–0.20 kg cap^−1^ week^−1^ on average), the average amount of kitchen and other organic waste collected via bio-waste bins is substantially lower in building C (0.31 kg cap^−1^ week^−1^) than in buildings A and B (0.66 and 0.71 kg cap^−1^ week^−1^; cf. Figure 2). However, in building C, bio-waste is also not collected via residual waste bins in comparably higher quantities (cf. Figure 3). In fact, the average amount of bio-waste collected via bio-waste bins and residual waste bins (1.08 kg cap^−1^ week^−1^) is the lowest of all three buildings (14% and 27% less than in buildings A and B, respectively), implying that substantial shares of bio-waste are either disposed of via other pathways or that the residents of building C generate less bio-waste per capita than those of buildings A and B.

#### Impurity contents of bio-waste bins

**Figure 5.**
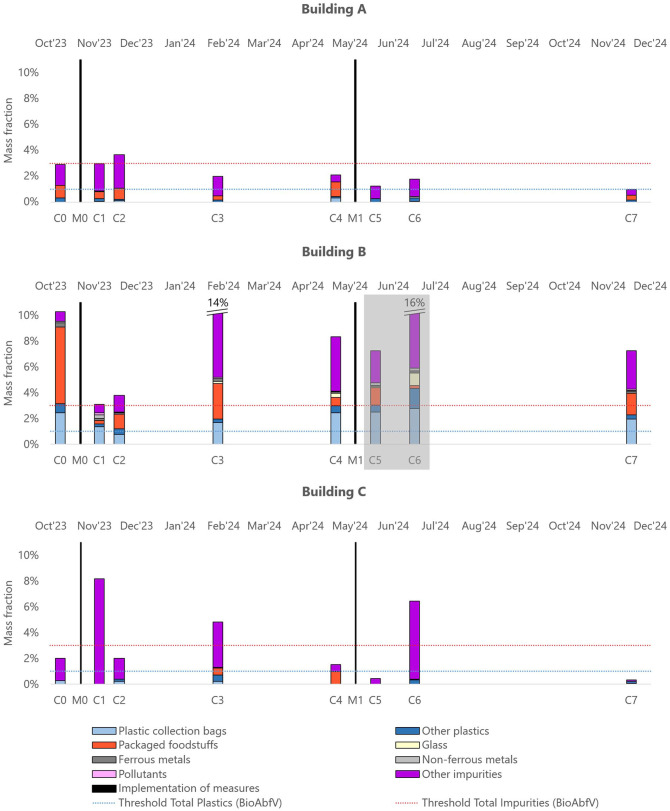
Contents of impurities in separately collected bio-wastes compared to thresholds set by the German Bio-Waste Ordinance (BioAbfV; Bundestag, 2022). C5 and C6 for building B (shaded area) must be considered with caution because of irregular bio-waste collection prior to the campaigns. C0–C7: waste characterization campaigns; M0, M1: implementation of measures.

Across all eight waste characterization campaigns, target materials made up 98%, 92% and 96% of separately collected bio-wastes from buildings A, B and C, respectively (cf. Figure 2), whereas non-target materials (impurities) accounted for the remaining part. As the overall impurity contents are very similar to those determined at the start of the investigation period (3%, 10% and 2% in buildings A, B and C, respectively, cf. Figure 5), the measures can be attested to have only little to no long-term impacts on the quality of separately collected bio-wastes. After implementing the measures (M0), the impurity content of separately collected bio-waste from building A intermediately increased to 4%, but showed a declining trend towards 1% in the further course of the investigation. In building B, impurities decreased to 3–4% after M0 but later on experienced a renewed increase to 7–16%. Here, plastic collection bags could partly, but not to the full extent, be displaced by paper collection bags. Similar to building B but at different levels, the impurity contents of separately collected bio-waste from building C ranged between <1% and 8% after M0. For both buildings B and C, particularly high contents of impurities can partly be traced back to certain events such as the disposal of single heavy items (e.g. building C, C1: bag of charcoal; building B, C6: bags of cat litter) or the disposal of a large number of similar items (e.g. building B, C0: packaged foodstuffs). Generally, impurities found in separately collected bio-waste could mostly be assigned to the characterization categories plastic collection bags, other plastics (primarily plastic packaging), packaged foodstuffs and others (primarily containing composite packaging and animal litter). Glass, metals and polluting/hazardous waste played a subordinate role in the vast majority of bio-wastes characterized.

### Self-perceived bio-waste separation behaviour

Even though 13% of the first survey’s participants (conducted before implementing the measures) had moved to Kassel less than 2 years previously, thus having higher potential to lack knowledge about the local waste collection scheme, all 24 participants declared that they felt rather informed (13) or very informed (11) about source separation of household wastes in general. Furthermore, all participants were aware that bio-waste should be collected separately. At the same time, four participants (17%; two households of buildings B and C, respectively) stated to only partly separate their household waste and to not have a separate bin for bio-waste collection in their household. Inconvenience (‘too little waste generation to make separation worthwhile’, ‘too many waste fractions to separate into’), lack of knowledge, demotivation due to poor source separation of other residents and hygiene concerns (especially in summer) were named as impediments for better source separation of bio-waste at household level.

During the second survey, residents were asked to what degree they felt the measures implemented had affected their bio-waste separation behaviour. Again, site-specific differences can be identified: while residents of building A perceived that handing out pre-sorting bins and hanging up the (first) poster influenced their bio-waste separation behaviour the most, residents of building B felt most affected by the combination of pre-sorting bins and paper collection bags, whereas residents of building C felt that conducting waste consultations and handing out multilingual information leaflets had the highest effects. The postal letter sent out mid-way through the investigation period as well as the second poster (showing the solution to the quiz on the first poster) were generally not recognized as influencing waste separation behaviour. Generally, participants from building A opined that the measures did rather not have a long-lasting impact on their bio-waste separation behaviour, while participants from buildings B and C reported a lasting effect of the measures.

## Discussion

### Effectiveness of measures implemented to foster the separate collection of bio-waste in MSRBs

#### Effects on the quantity of separately collected bio-waste

The SCRs of buildings investigated in this study were already relatively high from the start (42–55% vs. an average of 24% for MSRBs >10 households in Kassel) and rather comparable to that of smaller, less anonymous MSRBs in Kassel (3–10 households: 47%; cf. Bachmann et al., 2025). A potential explanation is that, over the years, Kassel’s municipal waste authority has withdrawn bio-waste bins across the city in the case of repeatedly unacceptable impurity levels, which led to many MSRBs (>10 households) not having access to bio-waste bins anymore, resulting in low overall SCRs for MSRBs (>10 households) in Kassel. At SCRs of 48–64% across all eight waste characterization campaigns, the investigated buildings rank within the upper half of the total range of SCRs for kerbside collection across Kassel (24–72%; Bachmann et al., 2025), and the increase in SCRs throughout the investigation period shows that the set of measures implemented was suitable to improve the separate collection of bio-waste in terms of quantity. As determined in previous studies, providing for convenient conditions at both household level (e.g. sorting equipment; cf. Bernstad, 2014) and building level (e.g. distance to collection points; cf. Rousta et al., 2017; Stoeva and Alriksson, 2017) is crucial for a high degree of bio-waste separation at source. Handing out pre-collection bins and compatible paper collection bags aimed exactly at facilitating bio-waste collection at household level and increased SCRs suggest that these two measures had the intended effect.

#### Effects on the quality of separately collected bio-waste

Regarding the quality of separately collected bio-waste, however, lasting impacts could neither be achieved by providing sorting equipment nor by interacting with residents through verbal (waste consultation), written (multilingual leaflet, letter) or visual (posters) communication. This is in line with results by Adam et al. (2024), who found that measures to change people’s bio-waste separation behaviour towards lower impurity levels were more effective when including the threat of a monetary penalty, whereas purely motivational approaches were not successful. Only in building A was a reduction in the total impurity contents of separately collected bio-wastes to slightly below 2% achieved. Starting at 3%, though, the level of impurities in building A was already relatively low. As reported by residents during waste consultations and interviews, one household had dedicated themselves to keeping the building’s bio-waste bins free of impurities by calling attention to wastes improperly disposed of by putting up photographs in the hallway. Cheng et al. (2024) determined that such behavioural traces are a significantly motivating factor for both waste separation intention and behaviour, and that waste separation intention is particularly positively affected by the feeling of guilt. Hereby, not just experienced guilt (being the denounced polluter) but also anticipated guilt (fearing to become the denounced polluter) comes into play (cf. Hurst and Sintov, 2022). Regarding building C, total impurity contents fell below the initial content of 2% three times but also surpassed it just as often, in these cases clearly exceeding the threshold of 3% set by the German Bio-Waste Ordinance (Bundestag, 2022; cf. Figure 5). Due to the overall low amounts of separately collected bio-waste in building C, single heavy items or a batch of material improperly disposed of via bio-waste bins had a relatively strong influence on the composition. Building B showed extraordinarily high levels of impurities (up to 16%) with no clear trend becoming evident. Due to the latest amendment to the German Bio-Waste Ordinance granting treatment plant operators the right to refuse treatment of household bio-wastes exceeding total impurity contents of 3% at delivery, batches evincing such poor quality are likely to be rejected and will have to be incinerated.

In buildings A and C, separately collected bio-waste was mostly collected either bagless or in paper collection bags (already from the start, cf. Supplemental Material), and the occurrence of bio- and non-degradable plastic collection bags remained the exception throughout the entire investigation period. In line with this, the total plastics threshold of 1% stipulated by the German Bio-Waste Ordinance (Bundestag, 2022) was never exceeded (cf. Figure 5). On the contrary, in building B, 70% of separately collected bio-waste were collected in plastic collection bags before the implementation of measures (cf. Supplemental Material). After implementation, a substantial decrease to 25% and 29% was determined during characterization campaigns C1 and C2, respectively, and besides an increase in bagless collection of gardening waste, plastic collection bags were partly replaced by the paper collection bags provided. Later, however, the amount of bio-waste separately collected in paper bags decreased and again, plastic collection bags were used instead. This implies that residents of building B were partly willing to change their bio-waste separation behaviour, but at the same time unwilling to purchase paper collection bags after using up the ones provided for free. Potentially, residents also perceived an increased feeling of surveillance after the implementation of measures, which diminished in the further course of the investigation period. Similarly, in their analyses of separately collected bio-waste from Austrian households, Adam et al. (2024) found relevant quantities of paper collection bags only if these were actively and continuously promoted. Due to their limited displacement in building B, the total plastics threshold of 1% was exceeded by the presence of plastic collection bags alone in seven out of eight characterization campaigns (cf. Figure 5), with plastic collection bags accounting for 64–86% of the total plastics contamination. Providing paper collection bags to displace plastic collection bags can therefore not be confirmed as an effective measure to reduce the plastic content of separately collected bio-waste.

### Concordance of observed and self-perceived bio-waste separation behaviour

Only building A, in which all survey participants declared to have good knowledge of bio-waste separation and to own a separate bio-waste pre-collection bin, provided separately collected bio-waste of constantly acceptable quality. Several residents of buildings B and C admitted to only partly separating their household waste and to not have a separate bin for bio-waste collection in their household, despite self-reporting good knowledge about the source separation of household wastes in general as well as bio-waste in particular. This is confirmed by the quality of separately collected bio-wastes, which displayed impurity contents of up to 16% and 8% in buildings B and C, respectively. For these residents, who are unwilling to improve their behaviour even after raising additional awareness during waste consultations about the consequences of impurities in bio-waste, access to the bio-waste bins should be withdrawn, as the disposal of mixed wastes (even if primarily consisting of bio-waste) can potentially ruin entire batches of bio-waste due to the limited technical and economical capabilities to reduce impurity contents after the collection stage (cf. Alessi et al., 2020; Friege and Eger, 2022). This could be achieved by putting a lock on bio-waste bins (already common practice in Kassel to prevent unauthorized access) and handing out keys solely to residents truly interested in separate bio-waste collection. While this may cause a quantitative decrease, a better quality of separately collected bio-waste and subsequent treatment products is to be expected. In the case of biological treatment of bio-waste, the recovery of high-quality products should be prioritized over mere mass-based recycling rates, as the environmental performance of biological treatment options greatly depends on the efficient use of compost and digestate (e.g. to substitute for mineral fertilizers; Bachmann et al., 2025), which can only be achieved if the quality demands of consumers are met.

Survey participants of building B stated that they felt the most affected in their bio-waste separation behaviour by the combination of pre-sorting bins and paper collection bags. When determining the status quo of bio-waste collection at the start of the investigation (C0), building B displayed the highest amounts of separately collected bio-waste collected in plastic collection bags across all three buildings (70% vs 1% and 11% in buildings A and C, respectively; cf. Supplemental Material). Within the first 4 weeks after the implementation of the measures (C1 and C2), a shift from plastic collection bags to paper collection bags was observed, together with a substantial decrease in both total plastics and total impurities content, confirming the residents’ self-perception. However, a decline in this replacement effect could already be identified in campaign C3 (14 weeks after M0), presumably because the paper collection bags provided for free had been used up. As the residents themselves perceived the provision of paper collection bags to be effective for a more efficient separate collection of bio-waste, handing them out on a regular basis might serve as a constant reminder and motivation for people to participate in the separate collection of bio-waste while at the same time enabling a convenient routine at household level (cf. Bernstad, 2014; Pedersen and Manhice, 2020). The fact that residents of buildings A and C felt more affected by pre-sorting bins and posters (relating to convenience, hygiene and awareness (cf. Bernstad, 2014; Stoeva and Alriksson, 2017) as well as waste consultations and multilingual information leaflets (relating to system trust and awareness (cf. Pedersen and Manhice, 2020; Rousta et al., 2017)), respectively, implies that campaigns promoting the separate collection of bio-waste should not aim at implementing a uniform set of measures but should rather be specifically designed to meet local predispositions and needs. Sewak et al. (2021) pointed out that behavioural intervention campaigns aiming to influence household-level waste sorting often lack consumer-oriented approaches and suggested to conduct formative research to generate insights from the residents. However, as shown by the low household coverage of this study’s survey, direct interaction with residents is difficult. Alternatively, upcoming AI-based impurity detection systems could come into effect, allowing for large-scale evaluation of poor-quality hotspots and predominant impurities ‘on the fly’ during municipal waste collection service – information which could in turn be used to customize measures.

### Limitations

During the waste characterization campaigns, the bio-waste bins and residual waste bins of all three buildings had to be collected on the same day for logistical reasons, partly resulting in shorter collection intervals compared to the regular municipal waste collection schedule (cf. Supplemental Material for exact intervals). Unlike for single family houses, in which residents are directly responsible for putting out waste bins on collection day and are likely to fill up empty bin volume, residents of multi-family buildings are usually not responsible for putting out waste bins and thus supposedly take out their waste depending on the filling level of the pre-collection bin within the household rather than orienting disposal behaviour in terms of the day of municipal waste collection. Influences of varying time intervals between waste collection during characterization campaigns and regular municipal waste collection are thus considered to be negligible.

On average, survey participants from buildings A, B and C lived in households with a size of 2.3 ± 1.1, 2.9 ± 0.7 and 2.0 ± 0.9 people, respectively (cf. Supplemental Material). Compared to mean household sizes based on the building characteristics given in Table 1 (2.8, 2.2 and 1.4 residents per household in buildings A, B and C, respectively), the survey covered below-average household sizes in building A, indicating that families and/or flat sharing communities might be underrepresented in the survey results, and above-average household sizes in buildings B and C, implying that single-households might be underrepresented. The overall survey participation rate of 38% of the households (despite repeated attempts to increase coverage in the buildings investigated) and the limited overlap between respondents of the first and second survey did not allow for a panel study regarding perceived information and behaviour throughout the investigation period.

As described in the literature and partly confirmed in this study, (bio-)waste separation behaviour depends on numerous factors such as hygiene, convenience, knowledge, attitude towards waste management, socio-demographic aspects, and social pressure (cf. Bernstad, 2014; Pedersen and Manhice, 2020; Rousta et al., 2017; Stoeva and Alriksson, 2017). Thus, despite the similar properties of the buildings investigated in this study (3× same number of households, 2× same number of storeys, 2× same city district), they differed with respect to collected amounts, impurity contents and SCRs of bio-waste, indicating the difficulty of transferring field study results from one building to another. As the evaluation of the study results is limited to descriptive statistics, future research should be based on a bigger sample size to enable the incorporation of inferential statistics in order to draw generalizable conclusions on the effectiveness of specific measures to increase the separate collection of bio-waste from MSRBs. Furthermore, the MSRBs to be investigated should be selected randomly rather than purposively, as the selection criteria used in this study (i.e. a similar number of households (>10), exclusive use of bio-waste bins by the residents, locations in different parts of the city, logistical considerations) might have introduced a bias affecting the transferability of conclusions to other MSRBs.

## Conclusion

By conducting eight waste characterization campaigns in combination with two series of structured interviews, the goal of this study was to evaluate the effectiveness of a set of measures regarding its suitability to increase the separate collection of bio-waste in MSRBs. The measures implemented (waste consultation, household sorting equipment, multilingual leaflet, letter, posters) were primarily suited to increase the quantity of separately collected bio-waste, while effects on the impurity contents varied across the three buildings investigated. The combination of observed effects on separate bio-waste collection and qualities with survey response patterns suggests that restricted access to bio-waste bins for dedicated households only might be an effective option to reduce impurity levels. The residents’ self-perception of the effectiveness of individual measures varied across the three buildings, indicating a need for site-specific sets of measures instead of uniform campaigns. Local issues must be tackled to motivate people to participate in the source separation of bio-waste. Furthermore, it could be shown that an intensified and sustained feeling of social responsibility and monitoring positively affected bio-waste separation behaviour. Municipal waste management authorities should therefore actively involve local actors such as dedicated residents, janitors or housing associations in their campaigns to increase quantities and qualities of separately collected urban bio-waste.

## Supplemental Material

sj-pdf-1-wmr-10.1177_0734242X261419233 – Supplemental material for Observation versus self-perception: Effectiveness of measures to increase separate bio-waste collection from multi-storey residential buildings Supplemental material, sj-pdf-1-wmr-10.1177_0734242X261419233 for Observation versus self-perception: Effectiveness of measures to increase separate bio-waste collection from multi-storey residential buildings by Konstantin Bachmann, Malek Simon Grimm, Ralf Wagner and David Laner in Waste Management & Research
