# Supplementary material for: Observation versus self-perception: Effectiveness of measures to increase separate bio-waste collection from multi-storey residential buildings
Source: Waste Manag Res. 2026 Feb 20;44(7):973–84. doi: 10.1177/0734242X261419233 (PMC13279979; doi:10.1177/0734242X261419233)

Supplementary Material

for

Observation vs. self-perception: Effectiveness of measures to increase separate bio-waste collection from multi-storey residential buildings

Konstantin Bachmann [a, *], Malek Simon Grimm [b], Ralf Wagner [b], David Laner [a]

[a] Research Group on Resource Management and Solid Waste Engineering, Faculty of Civil and Environmental Engineering, University of Kassel, Mönchebergstraße 7, 34125, Kassel, Germany

[b] Chair of Sustainable Marketing, Faculty of Economics and Management, University of Kassel, Henschelstraße 2, 34127 Kassel, Germany

* Corresponding author (e-mail address: k.bachmann@uni-kassel.de)

This file contains information on the waste characterization campaigns (sorting categories, detailed results), the household surveys (questions, responses), the waste collection intervals, the location of the buildings investigated, and pictures of the measures implemented.

Contents

Bio-waste characterization campaigns 3

Residual waste characterization campaigns 7

Household survey I 13

Household survey II 19

Waste collection intervals 28

Pictures of the measures implemented 29

Location of the buildings investigated 30

Bio-waste - Building A

Bio-waste (> 40 mm)

		Campaign	C0	C1	C2	C3	C4	C5	C6	C7
Level I	Level II	Level III (Sorting categories)	Mass [kg]							
Impurities	Plastic collection bags	Plastic collection bags	0.000	0.058	0.167	0.000	0.340	0.000	0.049	0.000
	Other plastics	Plastic packaging	0.034	0.211	0.075	0.137	0.099	0.110	0.202	0.074
		Other plastic products	0.139	0.019	0.029	0.005	0.057	0.119	0.118	0.013
	Glass	Glass packaging	0.000	0.000	0.000	0.000	0.000	0.000	0.000	0.000
		Other glass products	0.000	0.000	0.000	0.000	0.000	0.000	0.000	0.000
	Ferrous metals	Ferrous metal packaging	0.000	0.008	0.000	0.000	0.000	0.000	0.000	0.000
		Other ferrous metal products	0.000	0.000	0.000	0.000	0.000	0.000	0.000	0.000
	Non-ferrous metals	Non-ferrous metal packaging	0.002	0.000	0.001	0.000	0.000	0.001	0.010	0.000
		Other non-ferrous metal products	0.000	0.051	0.027	0.000	0.000	0.000	0.074	0.000
	Packaged foodstuffs	Packaged foodstuffs	0.866	0.634	1.164	0.373	1.302	0.000	0.000	0.494
	Pollutants	Pollutants	0.000	0.020	0.000	0.000	0.000	0.000	0.000	0.000
	Other impurities	Minerals	0.000	0.000	0.000	0.000	0.000	0.000	0.000	0.000
		Textiles	0.000	0.010	0.159	0.003	0.000	0.000	0.000	0.000
		Others	0.842	1.280	2.949	1.450	0.593	0.763	1.521	0.344
Target materials	Gardening waste	Gardening waste	3.142	2.644	9.644	5.203	4.622	5.235	9.588	24.349
	Kitchen and other organic waste	Kitchen and other organic waste	37.574	50.412	62.967	50.975	41.045	35.545	40.245	45.031
	Paper collection bags	Paper collection bags	0.241	1.956	1.508	1.907	2.703	1.273	1.783	2.240
Particles < 40 mm	Particles < 40 mm	Particles < 40 mm	46.712	61.181	57.017	76.414	64.472	65.356	63.216	65.335
Sum			89.552	118.484	135.707	136.467	115.233	108.402	116.806	137.880

Bio-waste (< 40 mm)

		Campaign	C0	C1	C2	C3	C4	C5	C6	C7
Level I	Level II	Level III (Sorting categories)	Mass [kg]							
Impurities	Plastic collection bags	Plastic collection bags	0.000	0.000	0.000	0.000	0.000	0.000	0.000	0.000
	Other plastics	Plastic packaging	0.008	0.001	0.000	0.005	0.000	0.002	0.001	0.001
		Other plastic products	0.000	0.000	0.000	0.000	0.001	0.000	0.000	0.007
	Glass	Glass packaging	0.000	0.000	0.000	0.000	0.000	0.000	0.000	0.000
		Other glass products	0.000	0.000	0.000	0.000	0.000	0.000	0.000	0.000
	Ferrous metals	Ferrous metal packaging	0.000	0.000	0.000	0.000	0.000	0.000	0.000	0.000
		Other ferrous metal products	0.000	0.000	0.000	0.000	0.000	0.000	0.000	0.000
	Non-ferrous metals	Non-ferrous metal packaging	0.000	0.000	0.000	0.000	0.000	0.000	0.000	0.000
		Other non-ferrous metal products	0.000	0.000	0.000	0.000	0.000	0.000	0.000	0.003
	Packaged foodstuffs	Packaged foodstuffs	0.000	0.000	0.000	0.000	0.000	0.000	0.000	0.000
	Pollutants	Pollutants	0.000	0.000	0.000	0.000	0.000	0.000	0.000	0.000
	Other impurities	Minerals	0.000	0.000	0.000	0.026	0.000	0.000	0.000	0.000
		Textiles	0.000	0.000	0.000	0.000	0.000	0.000	0.000	0.000
		Others	0.044	0.080	0.022	0.005	0.001	0.016	0.008	0.018
Target materials	Gardening waste	Gardening waste	0.035	0.111	0.118	0.149	0.081	0.246	0.157	0.161
	Kitchen and other organic waste	Kitchen and other organic waste	2.902	3.253	1.978	2.864	3.588	2.883	4.179	3.788
	Paper collection bags	Paper collection bags	0.000	0.000	0.000	0.004	0.000	0.000	0.000	0.000
	Particles < 10 mm	Particles < 10 mm	0.375	0.729	1.113	0.656	0.265	0.584	0.154	0.541
Sum			3.364	4.174	3.231	3.709	3.936	3.731	4.499	4.519

Bio-waste - Building B

Bio-waste (> 40 mm)

		Campaign	C0	C1	C2	C3	C4	C5	C6	C7
Level I	Level II	Level III (Sorting categories)				Mass [kg]				
Impurities	Plastic collection bags	Plastic collection bags	1.357	1.215	0.588	1.659	5.072	5.526	3.420	1.304
	Other plastics	Plastic packaging	0.247	0.159	0.182	0.170	0.759	1.047	0.972	0.094
		Other plastic products	0.133	0.010	0.097	0.032	0.300	0.023	0.839	0.133
	Glass	Glass packaging	0.000	0.000	0.000	0.000	0.452	0.344	0.682	0.000
		Other glass products	0.000	0.000	0.000	0.169	0.229	0.000	0.267	0.000
	Ferrous metals	Ferrous metal packaging	0.175	0.161	0.085	0.169	0.137	0.122	0.256	0.115
		Other ferrous metal products	0.000	0.000	0.000	0.013	0.000	0.000	0.000	0.000
	Non-ferrous metals	Non-ferrous metal packaging	0.018	0.204	0.000	0.025	0.104	0.070	0.002	0.026
		Other non-ferrous metal products	0.016	0.013	0.023	0.016	0.068	0.197	0.188	0.025
	Packaged foodstuffs	Packaged foodstuffs	3.287	0.259	0.833	2.688	1.393	2.855	0.267	1.048
	Pollutants	Pollutants	0.000	0.110	0.000	0.000	0.011	0.000	0.000	0.042
	Other impurities	Minerals	0.000	0.000	0.000	0.000	0.158	0.000	0.000	1.153
		Textiles	0.000	0.000	0.094	0.136	0.148	0.179	0.560	0.037
		Others	0.378	0.544	0.784	8.657	8.236	4.360	12.108	0.792
Target materials	Gardening waste	Gardening waste	5.623	39.737	4.244	14.722	25.422	28.150	0.500	9.378
	Kitchen and other organic waste	Kitchen and other organic waste	21.044	21.512	30.745	43.262	66.471	61.480	37.945	24.213
	Paper collection bags	Paper collection bags	0.247	0.956	0.881	1.643	2.991	4.152	1.017	0.445
Particles < 40 mm	Particles < 40 mm	Particles < 40 mm	23.079	25.371	35.434	24.414	96.964	111.425	64.216	27.623
Sum			55.604	90.251	73.990	97.775	208.915	219.930	123.239	66.428

Bio-waste (< 40 mm)

		Campaign	C0	C1	C2	C3	C4	C5	C6	C7
Level I	Level II	Level III (Sorting categories)				Mass [kg]				
Impurities	Plastic collection bags	Plastic collection bags	0.000	0.000	0.000	0.000	0.000	0.000	0.000	0.000
	Other plastics	Plastic packaging	0.004	0.001	0.003	0.008	0.005	0.008	0.009	0.000
		Other plastic products	0.000	0.002	0.000	0.001	0.000	0.000	0.001	0.000
	Glass	Glass packaging	0.000	0.000	0.000	0.000	0.000	0.000	0.000	0.000
		Other glass products	0.000	0.000	0.000	0.000	0.000	0.000	0.019	0.000
	Ferrous metals	Ferrous metal packaging	0.000	0.000	0.000	0.000	0.000	0.000	0.000	0.000
		Other ferrous metal products	0.000	0.000	0.000	0.000	0.000	0.000	0.000	0.000
	Non-ferrous metals	Non-ferrous metal packaging	0.008	0.000	0.001	0.000	0.000	0.005	0.002	0.001
		Other non-ferrous metal products	0.000	0.000	0.000	0.000	0.000	0.003	0.001	0.000
	Packaged foodstuffs	Packaged foodstuffs	0.000	0.000	0.000	0.000	0.000	0.000	0.000	0.005
	Pollutants	Pollutants	0.000	0.003	0.000	0.015	0.000	0.000	0.002	0.002
	Other impurities	Minerals	0.000	0.000	0.000	0.000	0.000	0.000	0.000	0.000
		Textiles	0.000	0.000	0.000	0.000	0.000	0.000	0.000	0.002
		Others	0.006	0.003	0.007	0.009	0.010	0.047	0.028	0.000
Target materials	Gardening waste	Gardening waste	0.012	0.011	0.028	0.003	0.070	0.009	0.005	0.000
	Kitchen and other organic waste	Kitchen and other organic waste	3.671	3.094	2.515	3.454	4.455	5.699	4.913	3.929
	Paper collection bags	Paper collection bags	0.000	0.000	0.000	0.000	0.000	0.000	0.000	0.000
	Particles < 10 mm	Particles < 10 mm	0.407	0.269	0.535	0.368	0.113	0.083	0.566	0.170
Sum			4.108	3.383	3.089	3.858	4.653	5.854	5.546	4.109

Bio-waste - Building C

Bio-waste (> 40 mm)

Level I	Level II	Level III (Sorting categories)	C0	C1	C2	C3	C4	C5	C6	C7
		Campaign	C0	C1	C2	C3	C4	C5	C6	C7
			Mass [kg]							
Impurities	Plastic collection bags	Plastic collection bags	0.075	0.000	0.109	0.065	0.000	0.000	0.000	0.022
	Other plastics	Plastic packaging	0.001	0.000	0.033	0.102	0.000	0.001	0.016	0.043
		Other plastic products	0.000	0.001	0.052	0.058	0.000	0.000	0.072	0.020
	Glass	Glass packaging	0.000	0.000	0.000	0.000	0.000	0.000	0.000	0.000
		Other glass products	0.000	0.000	0.000	0.000	0.000	0.000	0.000	0.000
	Ferrous metals	Ferrous metal packaging	0.000	0.000	0.000	0.000	0.000	0.000	0.000	0.000
		Other ferrous metal products	0.000	0.000	0.000	0.000	0.000	0.000	0.000	0.000
	Non-ferrous metals	Non-ferrous metal packaging	0.002	0.000	0.003	0.001	0.000	0.000	0.002	0.000
		Other non-ferrous metal products	0.000	0.000	0.000	0.000	0.000	0.000	0.000	0.000
	Packaged foodstuffs	Packaged foodstuffs	0.000	0.000	0.000	0.163	0.502	0.000	0.000	0.000
	Pollutants	Pollutants	0.000	0.000	0.007	0.000	0.000	0.000	0.000	0.000
	Other impurities	Minerals	0.000	0.000	0.289	0.000	0.000	0.110	0.032	0.000
		Textiles	0.000	0.000	0.000	0.007	0.000	0.000	0.000	0.000
		Others	0.304	2.840	0.488	1.085	0.164	0.034	1.273	0.027
Target materials	Gardening waste	Gardening waste	1.782	1.144	6.212	1.852	15.412	5.207	0.698	7.283
	Kitchen and other organic waste	Kitchen and other organic waste	11.233	16.012	21.112	11.917	10.545	7.790	5.635	14.192
	Paper collection bags	Paper collection bags	0.486	1.022	0.951	1.209	1.362	0.769	1.075	0.715
Particles < 40 mm	Particles < 40 mm	Particles < 40 mm	12.574	14.834	23.745	14.726	22.232	42.771	15.616	17.227
Sum			26.457	35.853	53.001	31.185	50.217	56.682	24.419	39.529

Bio-waste (< 40 mm)

Level I	Level II	Level III (Sorting categories)	C0	C1	C2	C3	C4	C5	C6	C7
		Campaign	C0	C1	C2	C3	C4	C5	C6	C7
			Mass [kg]							
Impurities	Plastic collection bags	Plastic collection bags	0.000	0.000	0.000	0.000	0.000	0.000	0.000	0.000
	Other plastics	Plastic packaging	0.000	0.000	0.000	0.000	0.000	0.000	0.000	0.000
		Other plastic products	0.000	0.001	0.000	0.000	0.000	0.001	0.000	0.000
	Glass	Glass packaging	0.000	0.000	0.000	0.000	0.000	0.000	0.000	0.000
		Other glass products	0.000	0.000	0.000	0.000	0.000	0.000	0.000	0.000
	Ferrous metals	Ferrous metal packaging	0.000	0.000	0.000	0.003	0.000	0.000	0.000	0.000
		Other ferrous metal products	0.000	0.000	0.000	0.000	0.000	0.000	0.000	0.000
	Non-ferrous metals	Non-ferrous metal packaging	0.000	0.000	0.001	0.000	0.000	0.000	0.000	0.000
		Other non-ferrous metal products	0.000	0.000	0.000	0.000	0.000	0.000	0.000	0.000
	Packaged foodstuffs	Packaged foodstuffs	0.000	0.000	0.000	0.000	0.000	0.000	0.000	0.000
	Pollutants	Pollutants	0.000	0.000	0.000	0.000	0.000	0.000	0.000	0.000
	Other impurities	Minerals	0.038	0.005	0.000	0.000	0.000	0.003	0.025	0.000
		Textiles	0.001	0.000	0.000	0.001	0.000	0.000	0.000	0.000
		Others	0.007	0.010	0.010	0.003	0.015	0.008	0.016	0.004
Target materials	Gardening waste	Gardening waste	0.133	0.180	0.214	0.040	0.110	0.103	0.096	0.047
	Kitchen and other organic waste	Kitchen and other organic waste	2.609	1.051	1.955	3.105	2.060	1.205	3.358	3.005
	Paper collection bags	Paper collection bags	0.000	0.000	0.000	0.000	0.000	0.000	0.000	0.000
	Particles < 10 mm	Particles < 10 mm	0.981	1.205	1.436	0.922	1.578	3.347	0.084	0.781
Sum			3.769	2.452	3.616	4.074	3.763	4.667	3.579	3.837

Bio-waste - Use of collection bags

Building A

Amounts of bio-waste collected…	C0	C1	C2	C3	C4	C5	C6	C7
				Mass [kg]				
in paper bags	5.886	22.590	22.256	24.749	13.049	15.821	14.856	13.591
in non-degradable plastic bags	0.706	0.524	3.072	0.000	0.853	0.000	0.769	0.000
in bio-degradable plastic bags	0.000	0.000	0.000	0.000	5.812	0.000	0.000	0.000
baglessly	82.960	95.370	110.379	111.718	95.519	92.581	101.181	124.289
Sum	89.552	118.484	135.707	136.467	115.233	108.402	116.806	137.880

Building B

Amounts of bio-waste collected…	C0	C1	C2	C3	C4	C5	C6	C7
				Mass [kg]				
in paper bags	1.677	12.546	13.122	17.961	29.087	31.821	5.945	4.777
in non-degradable plastic bags	31.915	19.534	19.761	48.675	108.559	92.888	75.917	38.942
in bio-degradable plastic bags	7.197	3.212	1.665	4.200	0.000	0.000	0.000	0.271
baglessly	14.815	54.959	39.442	26.939	71.269	95.221	41.377	22.438
Sum	55.604	90.251	73.990	97.775	208.915	219.930	123.239	66.428

Building C

Amounts of bio-waste collected…	C0	C1	C2	C3	C4	C5	C6	C7
				Mass [kg]				
in paper bags	5.745	11.626	10.040	8.784	8.849	8.756	6.532	3.228
in non-degradable plastic bags	2.961	0.000	2.544	2.276	0.000	0.000	0.000	0.191
in bio-degradable plastic bags	0.000	0.000	0.000	0.000	0.000	0.000	0.000	0.000
baglessly	17.751	24.227	40.417	20.125	41.368	47.926	17.887	36.110
Sum	26.457	35.853	53.001	31.185	50.217	56.682	24.419	39.529

Residual waste - Building A

Residual waste (> 40 mm)

	Campaign	C0	C1	C2	C3	C4	C5	C6	C7
Level I	Level II (Sorting categories)				Mass [kg]				
Ferrous metals	Ferrous metal packaging	0.797	0.787	0.815	0.523	0.644	0.146	0.851	0.813
	Other ferrous metal products	0.000	0.067	0.000	2.120	0.097	0.346	0.000	1.436
Non-ferrous metals	Non-ferrous metal packaging	0.115	0.124	0.343	0.281	0.366	0.170	0.278	0.198
	Other non-ferrous metal products	0.029	0.113	0.099	0.108	0.402	0.046	0.171	0.173
Paper and cardboard	Paper and cardboard packaging	3.775	6.483	4.046	4.643	5.192	3.406	3.937	7.279
	Print products	2.303	8.099	1.409	3.655	1.395	1.478	0.931	32.848
	Other paper and cardboard products	1.353	0.480	0.633	0.857	0.965	1.008	0.524	10.823
Glass	Glass packaging	4.059	7.385	4.785	3.421	6.244	2.240	3.180	6.002
	Hollow glassware	0.143	1.431	1.858	1.145	0.000	0.201	0.208	0.951
	Other glass products	0.000	0.000	0.000	0.000	0.192	0.000	0.000	0.615
Plastics	Plastic packaging	3.875	4.875	3.975	6.275	5.492	2.392	5.373	3.969
	Other plastic products	2.992	1.892	1.292	4.092	2.917	1.475	2.642	7.580
Bio-waste	Kitchen waste	25.244	15.112	13.912	15.245	24.345	9.545	19.145	16.276
	Gardening waste	0.544	0.244	0.202	0.363	1.922	0.091	1.386	0.958
	Other organic waste	0.093	0.287	0.024	0.067	0.050	0.059	0.040	0.050
Hygienic paper	Hygienic paper	10.551	8.651	6.729	10.085	8.985	5.610	9.150	9.688
Wood	Wood packaging	0.000	0.000	0.028	0.000	0.266	0.035	0.003	0.011
	Other wood products	0.034	1.519	6.148	2.892	0.492	0.809	19.300	0.319
Textiles	Apparel textiles	1.542	2.226	1.409	1.293	2.135	4.819	4.531	1.113
	Shoes	4.930	2.530	1.576	1.655	2.983	6.490	2.377	2.584
	Other textile products	0.215	0.000	0.408	0.752	0.908	0.436	0.965	0.000
Minerals	Ceramics and porcelain	1.731	0.622	1.113	0.624	3.673	0.630	2.129	0.839
	Other minerals	0.000	0.000	0.000	0.000	0.000	0.000	0.006	0.007
Composites	Composite packaging	1.756	2.812	2.143	3.028	3.350	1.593	2.361	2.550
	Electrical and electronic appliances	7.330	1.368	0.636	4.543	2.450	0.113	1.476	6.439
	Furniture	0.000	0.125	0.000	0.221	0.000	0.000	0.000	0.111
	Vehicle components	0.000	0.000	0.000	0.000	0.000	0.000	0.000	0.000
	Other composites	0.728	1.046	0.458	0.277	2.423	0.842	0.751	11.979
Pollutants	Batteries	0.047	0.128	0.623	0.000	0.042	0.000	0.000	0.146
	Accumulators	0.000	0.000	0.000	0.000	0.000	0.000	0.000	0.026
	Drugs	0.010	0.000	0.145	0.508	0.182	0.000	0.144	0.141
	Chemicals	0.000	0.000	0.000	1.070	0.000	0.000	0.000	0.000
	Waste oil containing materials	0.000	0.000	0.000	0.000	0.000	0.000	0.000	0.000
	Other pollutants	0.058	0.143	0.651	0.826	0.000	0.000	0.000	0.092
Others	Leather	0.000	0.130	0.030	0.039	0.000	0.000	0.000	0.938
	Rubber	0.315	0.193	1.244	0.167	0.126	0.080	0.068	0.527
	Cork	0.000	0.000	0.006	0.000	0.000	0.000	0.000	0.008
	Hygienic products	14.576	28.512	9.276	26.466	22.952	6.882	31.552	19.665
	Packaged goods	10.936	15.236	9.436	7.380	18.852	4.645	5.258	12.299
	Others	6.622	8.022	11.122	2.655	13.736	3.875	17.036	31.610
Particles < 40 mm	Particles < 40 mm	9.761	11.949	13.449	18.049	16.345	10.980	19.590	13.272
Sum		116.464	132.591	100.023	125.325	150.123	70.442	155.363	204.335

Residual waste - Building A

Residual waste (< 40 mm)

	Campaign	C0	C1	C2	C3	C4	C5	C6	C7
Level I	Level II (Sorting categories)				Mass [kg]				
Ferrous metals	Ferrous metals	0.024	0.002	0.017	0.064	0.014	0.017	0.024	0.020
Non-ferrous metals	Non-ferrous metals	0.004	0.002	0.002	0.002	0.001	0.001	0.018	0.016
Paper and cardboard	Paper and cardboard	0.100	0.063	0.021	0.027	0.019	0.025	0.026	0.029
Glass	Glass	0.000	0.010	0.012	0.033	0.002	0.003	0.002	0.000
Plastics	Plastics	0.018	0.058	0.027	0.064	0.053	0.023	0.023	0.038
Bio-waste	Bio-waste	0.881	0.700	0.843	1.373	1.040	1.447	1.727	1.264
Hygienic paper	Hygienic paper	0.027	0.000	0.031	0.067	0.020	0.171	0.129	0.049
Wood	Wood	0.023	0.003	0.020	0.025	0.011	0.010	0.006	0.006
Textiles	Textiles	0.005	0.005	0.001	0.001	0.001	0.002	0.003	0.004
Minerals	Minerals	0.000	0.044	0.000	0.000	0.008	0.000	0.013	0.000
Composites	Composites	0.086	0.131	0.011	0.022	0.031	0.012	0.049	0.016
Pollutants	Pollutants	0.043	0.044	0.001	0.014	0.000	0.007	0.000	0.069
Others	Others	0.049	0.089	0.065	0.110	0.119	0.031	0.066	0.209
Particles < 10 mm	Particles < 10 mm	0.421	1.148	0.628	0.736	0.566	0.726	0.699	0.793
Sum		1.681	2.299	1.679	2.538	1.885	2.475	2.785	2.513

Residual waste - Building B

Residual waste (> 40 mm)

Level I	Level II (Sorting categories)	C0	C1	C2	C3	C4	C5	C6	C7
	Campaign	C0	C1	C2	C3	C4	C5	C6	C7
		Mass [kg]							
Ferrous metals	Ferrous metal packaging	0.945	1.329	0.856	0.028	1.597	0.580	1.849	1.242
	Other ferrous metal products	3.466	0.001	0.134	2.379	0.727	0.000	0.038	1.118
Non-ferrous metals	Non-ferrous metal packaging	0.135	0.348	0.483	0.085	0.383	0.147	0.377	0.059
	Other non-ferrous metal products	0.008	0.106	0.256	0.000	0.040	0.111	0.249	0.204
Paper and cardboard	Paper and cardboard packaging	7.593	7.066	8.339	1.655	5.918	4.497	7.526	8.460
	Print products	3.599	2.599	4.901	0.175	1.494	9.257	0.988	6.131
	Other paper and cardboard products	0.239	0.280	1.856	0.116	0.969	1.151	0.131	0.736
Glass	Glass packaging	2.985	4.885	4.088	0.431	3.671	1.774	3.844	5.367
	Hollow glassware	0.437	0.057	1.407	0.000	0.000	0.000	0.218	0.395
	Other glass products	0.000	0.000	0.034	0.000	0.107	0.000	0.007	1.328
Plastics	Plastic packaging	6.775	7.375	5.775	1.816	7.433	4.237	7.071	5.071
	Other plastic products	4.792	1.868	4.392	0.521	3.375	1.075	3.971	5.927
Bio-waste	Kitchen waste	28.319	23.112	25.212	2.607	26.445	4.674	13.564	6.661
	Gardening waste	0.114	0.252	0.001	0.000	0.676	0.003	0.014	0.249
	Other organic waste	0.006	0.037	0.072	0.011	0.085	0.046	0.015	0.019
Hygienic paper	Hygienic paper	6.451	8.751	7.483	1.539	7.885	3.350	6.283	5.269
Wood	Wood packaging	0.009	0.102	0.022	0.001	0.000	0.000	0.008	0.006
	Other wood products	0.537	0.615	0.319	0.000	0.003	0.000	0.000	4.930
Textiles	Apparel textiles	0.630	0.460	1.269	0.000	1.393	1.336	1.092	1.086
	Shoes	20.760	0.598	0.905	0.293	0.279	1.950	0.815	5.155
	Other textile products	0.000	0.517	0.189	0.000	0.000	0.000	0.000	0.716
Minerals	Ceramics and porcelain	0.669	0.515	0.450	0.000	0.030	0.000	0.000	1.646
	Other minerals	0.507	0.000	0.000	0.000	0.000	0.111	0.254	0.000
Composites	Composite packaging	3.212	4.012	2.246	1.203	3.034	1.598	2.582	1.869
	Electrical and electronic appliances	2.136	7.630	0.952	0.000	0.346	0.000	0.194	0.273
	Furniture	0.000	7.628	0.000	0.000	1.831	0.000	0.000	4.320
	Vehicle components	0.000	0.000	0.000	0.000	0.000	0.000	0.000	0.000
	Other composites	2.028	3.628	0.601	0.000	1.162	0.095	0.204	1.543
Pollutants	Batteries	0.000	0.000	0.000	0.000	0.023	0.000	0.000	0.047
	Accumulators	0.000	0.000	0.000	0.000	0.000	0.000	0.000	0.000
	Drugs	0.000	0.000	0.007	0.000	0.014	0.000	0.013	0.005
	Chemicals	0.145	0.000	0.000	0.000	0.030	0.000	0.000	0.000
	Waste oil containing materials	0.000	0.000	0.000	0.000	0.000	0.000	0.042	0.000
	Other pollutants	1.290	0.373	0.034	0.000	0.000	0.000	0.000	0.016
Others	Leather	0.000	0.393	0.000	0.000	0.000	0.000	0.090	0.016
	Rubber	0.125	0.056	0.728	0.014	0.181	0.019	0.316	0.052
	Cork	0.000	0.000	0.000	0.000	0.000	0.000	0.000	0.000
	Hygienic products	15.376	11.276	9.676	3.520	17.766	7.815	13.559	13.140
	Packaged goods	10.436	21.736	5.822	1.417	13.634	3.093	9.180	6.642
	Others	0.734	1.977	3.472	0.098	7.636	11.641	7.166	10.861
Particles < 40 mm	Particles < 40 mm	13.261	20.334	13.390	6.044	29.152	7.044	18.489	9.743
Sum		137.719	139.916	105.371	23.953	137.319	65.604	100.149	110.302

Residual waste - Building B

Residual waste (< 40 mm)

	Campaign	C0	C1	C2	C3	C4	C5	C6	C7
Level I	Level II (Sorting categories)				Mass [kg]				
Ferrous metals	Ferrous metals	0.005	0.018	0.015	0.006	0.000	0.001	0.006	0.005
Non-ferrous metals	Non-ferrous metals	0.010	0.002	0.007	0.010	0.003	0.024	0.001	0.013
Paper and cardboard	Paper and cardboard	0.042	0.026	0.056	0.019	0.024	0.032	0.020	0.034
Glass	Glass	0.012	0.000	0.027	0.012	0.010	0.000	0.000	0.004
Plastics	Plastics	0.065	0.027	0.058	0.024	0.042	0.038	0.094	0.082
Bio-waste	Bio-waste	1.395	1.689	1.746	2.193	2.113	2.420	1.016	1.224
Hygienic paper	Hygienic paper	0.020	0.042	0.047	0.025	0.032	0.045	0.031	0.081
Wood	Wood	0.043	0.004	0.001	0.004	0.006	0.019	0.002	0.014
Textiles	Textiles	0.003	0.000	0.000	0.000	0.000	0.004	0.001	0.001
Minerals	Minerals	0.068	0.000	0.000	0.000	0.007	0.000	0.000	0.000
Composites	Composites	0.032	0.021	0.026	0.043	0.028	0.047	0.046	0.018
Pollutants	Pollutants	0.035	0.049	0.085	0.010	0.000	0.020	0.000	0.066
Others	Others	0.130	0.046	0.048	0.054	0.095	0.018	0.449	0.080
Particles < 10 mm	Particles < 10 mm	1.274	0.360	0.626	1.271	0.462	0.514	0.420	0.442
Sum		3.134	2.284	2.742	3.671	2.822	3.182	2.086	2.064

Residual waste - Building C

Residual waste (> 40 mm)

Level I	Level II (Sorting categories)	C0	C1	C2	C3	C4	C5	C6	C7
	Campaign	C0	C1	C2	C3	C4	C5	C6	C7
		Mass [kg]							
Ferrous metals	Ferrous metal packaging	0.385	2.478	0.956	0.206	0.408	0.200	0.095	0.035
	Other ferrous metal products	0.031	1.068	0.000	2.529	0.010	0.001	0.231	0.000
Non-ferrous metals	Non-ferrous metal packaging	0.062	0.170	0.148	0.139	0.263	0.097	0.159	0.002
	Other non-ferrous metal products	0.046	0.153	0.042	0.106	0.100	0.014	0.072	0.000
Paper and cardboard	Paper and cardboard packaging	1.683	2.083	1.528	1.704	3.327	1.728	1.381	0.458
	Print products	0.899	2.199	0.437	0.612	1.981	0.443	1.143	0.198
	Other paper and cardboard products	0.080	0.380	0.452	0.279	0.823	0.306	0.201	0.135
Glass	Glass packaging	1.285	4.385	2.822	1.440	1.389	0.448	1.687	0.481
	Hollow glassware	0.231	0.000	0.000	0.728	0.107	0.018	0.165	0.616
	Other glass products	0.000	4.127	0.000	0.000	0.000	0.536	0.043	0.000
Plastics	Plastic packaging	1.775	2.475	0.975	2.192	1.892	2.992	1.545	0.128
	Other plastic products	1.092	3.792	1.092	2.175	2.175	1.975	1.068	0.167
Bio-waste	Kitchen waste	11.744	13.612	8.412	15.938	11.845	7.902	6.691	1.662
	Gardening waste	0.031	0.844	0.197	0.232	7.722	0.206	0.251	0.933
	Other organic waste	0.003	0.005	0.003	0.008	0.025	0.016	0.015	0.017
Hygienic paper	Hygienic paper	4.051	5.251	3.997	5.790	6.085	2.758	3.609	0.559
Wood	Wood packaging	0.019	0.031	0.002	0.000	0.020	0.003	0.004	0.356
	Other wood products	6.930	7.530	0.007	0.005	9.400	0.000	1.309	0.000
Textiles	Apparel textiles	2.332	4.732	2.380	0.543	0.265	0.803	3.667	0.102
	Shoes	0.230	7.230	6.285	0.550	0.996	0.161	3.007	0.000
	Other textile products	0.675	11.061	1.944	0.090	1.307	0.914	0.000	0.000
Minerals	Ceramics and porcelain	0.407	0.734	4.820	0.734	0.881	1.878	1.089	0.000
	Other minerals	0.000	0.000	0.529	0.000	0.000	0.000	0.000	0.000
Composites	Composite packaging	0.656	1.156	0.645	1.012	1.587	0.503	0.495	0.039
	Electrical and electronic appliances	0.323	5.430	6.936	0.113	0.016	0.000	0.091	0.043
	Furniture	0.000	6.600	0.000	0.000	7.479	0.000	0.000	0.000
	Vehicle components	0.000	0.000	0.000	0.000	0.000	0.000	0.000	0.000
	Other composites	1.128	4.228	3.433	0.041	0.775	0.040	1.287	0.002
Pollutants	Batteries	0.024	0.000	0.011	0.000	0.000	0.000	0.000	0.000
	Accumulators	0.000	0.000	0.000	0.000	0.000	0.000	0.000	0.000
	Drugs	0.000	0.000	0.166	0.239	0.174	0.000	0.036	0.000
	Chemicals	0.000	0.212	0.000	0.000	0.000	0.073	0.117	0.000
	Waste oil containing materials	0.000	0.000	0.000	0.000	0.000	0.000	0.008	0.000
	Other pollutants	0.003	0.000	0.001	0.088	0.000	0.009	0.000	0.000
Others	Leather	0.000	0.000	0.028	0.000	0.000	0.000	0.000	0.000
	Rubber	0.026	0.172	0.199	0.036	0.047	0.076	0.011	0.035
	Cork	0.000	0.068	0.012	0.000	0.000	0.000	0.000	0.000
	Hygienic products	6.876	14.010	12.033	7.763	5.176	6.038	4.905	0.151
	Packaged goods	5.636	10.436	4.763	7.189	11.932	1.926	4.649	0.915
	Others	1.200	3.132	4.309	1.219	8.604	1.988	2.767	2.055
Particles < 40 mm	Particles < 40 mm	8.415	13.710	9.034	16.784	25.250	9.548	7.950	3.785
Sum		58.278	133.494	78.598	70.484	112.061	43.600	49.748	12.874

Residual waste - Building C

Residual waste (< 40 mm)

	Campaign	C0	C1	C2	C3	C4	C5	C6	C7
Level I	Level II (Sorting categories)				Mass [kg]				
Ferrous metals	Ferrous metals	0.008	0.013	0.013	0.004	0.000	0.009	0.024	0.092
Non-ferrous metals	Non-ferrous metals	0.001	0.002	0.002	0.002	0.039	0.002	0.001	0.001
Paper and cardboard	Paper and cardboard	0.011	0.024	0.031	0.097	0.031	0.016	0.025	0.016
Glass	Glass	0.013	0.006	0.004	0.004	0.006	0.002	0.012	0.009
Plastics	Plastics	0.052	0.339	0.069	0.027	0.032	0.003	0.017	0.010
Bio-waste	Bio-waste	1.596	1.500	1.335	1.045	1.265	1.727	1.112	0.983
Hygienic paper	Hygienic paper	0.023	0.066	0.037	0.069	0.042	0.045	0.064	0.025
Wood	Wood	0.006	0.013	0.006	0.001	0.005	0.003	0.014	0.006
Textiles	Textiles	0.004	0.000	0.006	0.001	0.000	0.001	0.002	0.000
Minerals	Minerals	0.000	0.000	0.071	0.000	0.000	0.000	0.000	0.000
Composites	Composites	0.034	0.037	0.050	0.031	0.037	0.024	0.004	0.018
Pollutants	Pollutants	0.018	0.010	0.051	0.053	0.000	0.009	0.007	0.119
Others	Others	0.034	0.099	0.115	0.243	2.089	0.028	0.066	0.019
Particles < 10 mm	Particles < 10 mm	0.769	0.552	0.818	1.056	1.955	0.821	0.785	2.353
Sum		2.569	2.661	2.608	2.633	5.501	2.690	2.133	3.651

Survey I

Abbreviation	Question
F1	To what extent are you informed about the source separation of household wastes: distribution to lightweight packaging waste, waste paper and cardboard, residual waste and bio-waste?
F2	Are you aware of what happens to your bio-waste after collection?
F3	How strictly do you separate your wastes?
F4	Are there specific reasons or aspects that hinder you from separately collecting your household wastes to a higher degree? If so, please state them.
F5	Are you aware of the obligation to collect bio-waste separately?
F6	Do you have a bin in your household in which you exclusively collect bio-waste?
F7	You just stated that you have a bin in your household in which you exclusively collect bio-waste. Do you use collection bags in this bin to collect your bio-waste?
F7a	Comment on F7
F8.1	To what extent would you agree with this statement? "Having to take out my bio-waste separately sometimes deters me from separating bio-waste from other wastes."
F8.2	To what extent would you agree with this statement? "Having to take out my bio-waste separately sometimes deters me from separating bio-waste from other wastes."
F9	The municipal waste authority provides an leaflet informing about proper source separation of wastes. Are you aware of this leaflet?
F10	Are you aware of the waste consultancy service of the municipal waste authority?
F11	How satisfied are you with the services provided by the municipal waste authority?
F12a	Why are you dissatisfied with the services provided by the municipal waste authority? Please state relevant aspects.
F12b	Why are you satisfied with the services provided by the municipal waste authority? Please state relevant aspects.
F12aa	How relevant are "sustainability" and "climate change" to you? -Sustainability in general
F12bb	How relevant are "sustainability" and "climate change" to you? -Climate change in general
F13a	Following this, we would like to know to what extent you see yourself as a person who lives sustainably and cares about climate change. -I am a person who lives sustainably.
F13b	Following this, we would like to know to what extent you see yourself as a person who lives sustainably and cares about climate change. -I am a person who cares about climate change.
F14	Are there specific reasons or aspects that hinder you from living more sustainably and climate friendly? If so, please state them.
S1	Please, state your age:
S2	Please, state your gender:
S3	Please, state your marital status:
S4	How many people live in your household?
S5	How many children under the age of 18 years live in your household?
S6	What is your highest educational degree?
S7	What is the total net income of your household?
S8	Are you affiliated with any religion? If so, which?
S8a	Comment on S8
S9	For how long have you been living in Kassel?
S10	How many times have you moved within Kassel?
S11	How many counties in Germany have you lived in - including Kassel?

Survey I

Abbreviation	Response options
F1	{1, Not informed at all}, {2 Rather not informed}, {3, Partly / partly}, {4, Rather informed}, {5, Well informed}
F2	{1, Not aware at all}, {2 Rather not aware}, {3, Partly / partly}, {4, Rather aware}, {5, Well aware}
F3	{1, Not strictly at all}, {2, Rather not strictly}, {3, Partly / partly}, {4, Rather strictly}, {5, Very strictly}
F4	Comment
F5	{1, No}, {2, Yes}
F6	{1, No}, {2, Yes}
F7	{1, No}, {2, Yes}
F7a	Comment
F8.1	{1, Strongly disagree}, {2, Rather disagree}, {3, Partly / partly}, {4, Rather agree}, {5, Strongly agree}
F8.2	{1, Strongly disagree}, {2, Rather disagree}, {3, Partly / partly}, {4, Rather agree}, {5, Strongly agree}
F9	{1, Yes}, {2, I'm not sure.}, {3, No}
F10	{1, Yes}, {2, I'm not sure.}, {3, No}
F11	{1, Very unsatisfied}, {2, Rather unsatisfied}, {3, Partly / partly}, {4, Rather satisfied}, {5, Very satisfied}
F12a	Comment
F12b	Comment
F12aa	{1, Very irrelevant}, {2, Rather irrelevant}, {3, Partly / partly}, {4, Rather relevant}, {5, Very relevant}
F12bb	{1, Very irrelevant}, {2, Rather irrelevant}, {3, Partly / partly}, {4, Rather relevant}, {5, Very relevant}
F13a	{1, Strongly disagree}, {2, Rather disagree}, {3, Partly / partly}, {4, Rather agree}, {5, Strongly agree}
F13b	{1, Strongly disagree}, {2, Rather disagree}, {3, Partly / partly}, {4, Rather agree}, {5, Strongly agree}
F14	Comment
S1	Comment
S2	{1, Female}, {2, Male}, {3, Non-binary}
S3	{1, Single}, {2, Married}, {3, Registered partnership}, {4, Divorced}, {5, Registered partnership nullified}, {6, Widowed}, {7, Registered partner deceased}
S4	Comment
S5	Comment
S6	{1, I prefer not to disclose}, {2, No school-leaving qualification}, {3, Still in school}, {4, Lower secondary education}, {5, Polytechnic secondary education}, {6, Secondary education}, {7, University of applied sciences entrance qualification} {8, General university entrance qualification}, {9, Apprenticeship completed}, {10, Master /Technician}, {11, Bachelor's degree (university of applied sciences)}, {12, Master's degree (university of applied sciences)}, {13, Bachelor's degree (university)}, {14, Master's degree (university)}, {15, Doctoral degree}
S7	{1, I prefer not to disclose}, {2, <1,000 €}, {3, 1,000 € - 1,999 €}, {4, 2,000 € - 2,999 €}, {5, 3,000 € - 3,999 €}, {6, 4,000 € - 4,999 €}, {7, 5,000 € - 5,999 €}, {8, 6,000 € - 6,999 €}, {9, ≥7,000 €}
S8	{1, I prefer not to disclose}, {2, No confession}, {3, Christianity, Catholic}, {4, Christianity, Protestant}, {5, Islam}, {6, Judaism}, {7, Hinduism}, {8, Buddhism}, {9, Others, that is …}
S8a	Comment
S9	{1, < 2 years}, {2, 2 years - <4 years}, {3, 4 years - <6 years}, {4, 6 years - <8 years}, {5, 8 years - <10 years}, {6, 10 years - <12 years}, {7, 12 years - <14 years}, {8, 14 years - <16 years}, {9, 16 years - <18 years}, {10, 18 years - <20 years}, {11, ≥20 years}
S10	{1, Not at all}, {2, Once}, {3, Twice}, {4, 3 times}, {5, 4 times}, {6, 5 times}, {7, >5 times}
S11	{1, 1}, {2, 2}, {3, 3}, {4, 4}, {5, 5}, {6, >5}

Survey I

Participant no	Building	F1	F2	F3	F4	F5	F6	F7	F7a	F8.1	F8.2	F9
1	C	5	2	5		2	2	1	Bin only	1	1	3
2	C	4	4	5	Information on multimaterials	2	2	1	Bin only	1	1	1
3	C	5	5	5		2	2	1	Mostly bin only, sometimes with bag	1	1	2
4	C	4	3	4		2	2	2		1	1	1
5	C	5	1	5	Multilayer packaging	2	2	1	Cardboard at the bottom	1	1	1
6	C	4	4	4	Multilayer packaging	2	2	1	Bin only	1	1	2
7	C	5	2	5	Hygiene	2	1			1	3	1
8	C	5	4	4	Too little waste (annoying)	2	1			5	1	1
9	A	5	4	5		2	2	1	Bin only	1	1	1
10	A	5	4	5		2	2	2		1	2	2
11	A	4	3	3		2	2	2		1	1	1
12	A	4	4	4		2	2	1	Bin only	1	3	1
13	A	4	3	4		2	2	1	Newspaper	3	3	1
14	A	5	2	5		2	2	1		1	4	1
15	A	4	4	3	Missing manufacturer specifications	2	2	2		2	3	1
16	A	5	1	5	Too many waste fractions to separate into	2	2	1	Sieve	1	4	1
17	A	5	1	5		2	2	2		1	3	1
18	A	4	1	4	Missing knowledge	2	2	1		1	1	1
19	B	4	1	4		2	2	2		1	1	2
20	B	4	1	3	Tidiness	2	1			4	3	3
21	B	5	3	4	Laziness	2	2	1		4	2	2
22	B	4	2	4		2	2	2		1	1	2
23	B	4	3	4	Waste separation behaviour of housemates	2	2	2		1	1	3
24	B	4	3	3		2	1			2	1	3
Mean	A	4.50	2.70	4.30	-	2.00	2.00	1.40	-	1.30	2.50	1.10
Standard deviation	A	0.53	1.34	0.82	-	0.00	0.00	0.52	-	0.67	1.18	0.32
Mean	B	4.17	2.17	3.67	-	2.00	1.67	1.75	-	2.17	1.50	2.50
Standard deviation	B	0.41	0.98	0.52	-	0.00	0.52	0.50	-	1.47	0.84	0.55
Mean	C	4.63	3.13	4.63	-	2.00	1.75	1.17	-	1.50	1.25	1.50
Standard deviation	C	0.52	1.36	0.52	-	0.00	0.46	0.41	-	1.41	0.71	0.76
Mean	Overall	4.46	2.71	4.25	-	2.00	1.83	1.40	-	1.58	1.83	1.58
Standard deviation	Overall	0.51	1.27	0.74	-	0.00	0.38	0.50	-	1.18	1.09	0.78

Survey I

Participant no	Building	F10	F11	F12a	F12b
1	C	1	9		Good and fast contact, no problems
2	C	2	8		Regular waste collection, enough bin capacity
3	C	2	8		Regular waste collection
4	C	2	8		Regular waste collection, waste paper and cardboard bins could be emptied more often
5	C	2	8		Generally satisfied
6	C	2	9		No negative aspects, fast, tidy
7	C	1	10		Waste collection on time
8	C	2	5	Bins are not put back in place after being emptied	
9	A	1	8		Reliable, good communication
10	A	2	9		Waste collection on time, even move bins from the collection points to the kerbside
11	A	1	6		Everything works fine
12	A	1	8		Reliable
13	A	2	10		Works well, capacity of waste paper and cardboard bins sometimes too little, good consulting
14	A	1	8		Friendly, fast, regular waste collection
15	A	2	8		No complications
16	A	2	7		Good service
17	A	2	8		Tidy, on time
18	A	2	9		No complications
19	B	2	6		Works well
20	B	2	9		Bin capacity is enough
21	B	2	6		Tidy, do their job
22	B	2	8		Nothing negative to report
23	B	2	8		Do their best, not being satisfied would be unfair
24	B	2	7		
Mean	A	1.60	8.10	-	-
Standard deviation	A	0.52	1.10	-	-
Mean	B	2.00	7.33	-	-
Standard deviation	B	0.00	1.21	-	-
Mean	C	1.75	8.13	-	-
Standard deviation	C	0.46	1.46	-	-
Mean	Overall	1.75	7.92	-	-
Standard deviation	Overall	0.44	1.25	-	-

Survey I

Participant no	Building	F12aa	F12bb	F13a	F13b	F14
1	C	4	5	4	4	Being unsatisfied with public transport
2	C	5	5	5	5	Laziness, lack of comfort
3	C	5	5	4	5	Pricing and packaging of foodstuffs
4	C	4	4	3	4	Time management
5	C	4	5	4	5	
6	C	5	5	3	3	Supply, infrastructure, laziness
7	C	5	5	4	3	Time management, lack of information, lack of transparency regarding policital targets
8	C	5	5	3	4	
9	A	4	5	4	4	
10	A	4	5	4	4	
11	A	3	3	4	4	
12	A	5	5	5	5	Pricing of organic and regional foodstuffs; pricing and development state of public transport
13	A	4	4	3	4	Finances, comfort, lack of time
14	A	4	4	3	3	Laziness
15	A	5	5	4	4	Unfavourable traffic routes, long distances
16	A	4	5	4	4	
17	A	4	2	4	4	
18	A	5	5	5	5	Travel
19	B	3	4	4	4	Laziness
20	B	4	5	4	4	Comfort
21	B	3	3	2	1	Unawareness, the feeling that small changes have no impact
22	B	4	3	3	4	
23	B	5	4	3	3	
24	B	3	3	3	3	
Mean	A	4.20	4.30	4.00	4.10	-
Standard deviation	A	0.63	1.06	0.67	0.57	-
Mean	B	3.67	3.67	3.17	3.17	-
Standard deviation	B	0.82	0.82	0.75	1.17	-
Mean	C	4.63	4.88	3.75	4.13	-
Standard deviation	C	0.52	0.35	0.71	0.83	-
Mean	Overall	4.21	4.33	3.71	3.88	-
Standard deviation	Overall	0.72	0.92	0.75	0.90	-

Survey I

Participant no	Building	S1	S2	S3	S4	S5	S6	S7	S8	S8a	S9	S10	S11
1	C	30	2	1	1	0	14	3	2		4	3	2
2	C	28	1	1	2	0	14	6	2		2	4	2
3	C	22	2	1	2	0	8	4	4		11	2	1
4	C	30	2	1	1	0	6	4	4		11	3	2
5	C	56	1	2	2	0	6	1	2		11	2	2
6	C	27	2	1	2	0	14	7	2		2	4	3
7	C	60	2	2	4	0	14	1	9	Christianity, Orthodox	11	3	3
8	C	90	1	1	1	0	8	1	4		11	4	4
9	A	71	2	2	2	0	1	1	1		11	7	1
10	A	27	2	2	4	2	14	6	2		5	2	2
11	A	84	2	6	1	0	14	6	3		11	2	1
12	A	59	1	1	3	0	14	1	1		11	5	1
13	A	45	1	2	5	3	8	5	4		11	5	1
14	A	81	2	2	2	0	14	7	3		8	1	6
15	A	74	1	1	1	0	14	1	2		11	4	4
16	A	74	1	4	2	0	15	7	4		11	2	6
17	A	71	1	2	2	0	14	5	4		11	3	1
18	A	70	2	2	2	0	14	5	4		7	7	3
19	B	25	2	1	4	0	8	1	2		3	3	4
20	B	21	2	1	4	0	8	6	4		11	2	1
21	B	35	1	4	3	2	6	3	5		11	6	2
22	B	26	2	1	2	0	7	5	2		11	6	3
23	B	24	2	1	3	0	4	1	2		11	7	1
24	B	33	1	1	3	1	4	4	3		1	1	3
Mean	A	65.60	1.50	2.40	2.40	0.50	12.20	4.40	2.80	-	9.70	3.80	2.60
Standard deviation	A	17.49	0.53	1.51	1.26	1.08	4.39	2.46	1.23	-	2.21	2.15	2.07
Mean	B	27.33	1.67	1.50	3.17	0.50	6.17	3.33	3.00	-	8.00	4.17	2.33
Standard deviation	B	5.47	0.52	1.22	0.75	0.84	1.83	2.07	1.26	-	4.69	2.48	1.21
Mean	C	42.88	1.63	1.25	1.88	0.00	10.50	3.38	3.63	-	7.88	3.13	2.38
Standard deviation	C	23.69	0.52	0.46	0.99	0.00	3.82	2.33	2.39	-	4.36	0.83	0.92
Mean	Overall	48.46	1.58	1.79	2.42	0.33	10.13	3.79	3.13	-	8.67	3.67	2.46
Standard deviation	Overall	23.49	0.50	1.25	1.14	0.82	4.33	2.28	1.68	-	3.64	1.88	1.50

Survey II

Abbreviation	Question
F0	Participated in first survey
F1	To what extent are you informed about the source separation of household wastes: distribution to lightweight packaging waste, waste paper and cardboard, residual waste and bio-waste?
F2	Are you aware of what happens to your bio-waste after collection?
F3	How strictly do you separate your wastes?
F4	Are there specific reasons or aspects that hinder you from separately collecting your household wastes to a higher degree? If so, please state them.
F5	Are you aware of the obligation to collect bio-waste separately?
F6	Do you have a bin in your household in which you exclusively collect bio-waste?
F7	You just stated that you have a bin in your household in which you exclusively collect bio-waste. Do you use collection bags in this bin to collect your bio-waste?
F7a	Comment on F7
F8.1	To what extent would you agree with this statement? "Having to take out my bio-waste separately sometimes deters me from separating bio-waste from other wastes."
F8.2	To what extent would you agree with this statement? "Having to take out my bio-waste separately sometimes deters me from separating bio-waste from other wastes."
F9	The municipal waste authority provides an leaflet informing about proper source separation of wastes. Are you aware of this leaflet?
F10	Are you aware of the waste consultancy service of the municipal waste authority?
F11	How satisfied are you with the services provided by the municipal waste authority?
F12a	Why are you dissatisfied with the services provided by the municipal waste authority? Please state relevant aspects.
F12b	Why are you satisfied with the services provided by the municipal waste authority? Please state relevant aspects.
F12aa	How relevant are "sustainability" and "climate change" to you? -Sustainability in general
F12bb	How relevant are "sustainability" and "climate change" to you? -Climate change in general
F13a	Following this, we would like to know to what extent you see yourself as a person who lives sustainably and cares about climate change. -I am a person who lives sustainably.
F13b	Following this, we would like to know to what extent you see yourself as a person who lives sustainably and cares about climate change. -I am a person who cares about climate change.
F14	Are there specific reasons or aspects that hinder you from living more sustainably and climate friendly? If so, please state them.
F15a	To which degree did you feel affected in your bio-waste separation behaviour by the following measure: Waste consultation
F15b	To which degree did you feel affected in your bio-waste separation behaviour by the following measure: Multilingual information leaflet
F15c	To which degree did you feel affected in your bio-waste separation behaviour by the following measure: Pre-collection bin
F15d	To which degree did you feel affected in your bio-waste separation behaviour by the following measure: Paper collection bags
F15e	To which degree did you feel affected in your bio-waste separation behaviour by the following measure: Postal letter
F15f	To which degree did you feel affected in your bio-waste separation behaviour by the following measure: Poster I
F15g	To which degree did you feel affected in your bio-waste separation behaviour by the following measure: Poster II
F16	To which degree did the implemented measures have a sustained, long-term impact on your waste separation behaviour?
F17a	Did the implemented measures change your attitude towards waste separation? If so, in which way?
F17b	Did the implemented measures change your behaviour regarding other aspects but waste separation (e.g., diet, traffic, shopping)?
S1	Please, state your age:
S2	Please, state your gender:
S3	Please, state your marital status:
S4	How many people live in your household?
S5	How many children under the age of 18 years live in your household?
S6	What is your highest educational degree?
S7	What is the total net income of your household?
S8	Are you affiliated with any religion? If so, which?
S8a	Comment on S8
S9	For how long have you been living in Kassel?
S10	How many times have you moved within Kassel?
S11	How many counties in Germany have you lived in - including Kassel?

Survey II

Abbreviation	Answer options
F0	{0, No}, {1, Yes}
F1	{1, Not informed at all}, {2 Rather not informed}, {3, Partly / partly}, {4, Rather informed}, {5, Well informed}
F2	{1, Not aware at all}, {2 Rather not aware}, {3, Partly / partly}, {4, Rather aware}, {5, Well aware}
F3	{1, Not strictly at all}, {2, Rather not strictly}, {3, Partly / partly}, {4, Rather strictly}, {5, Very strictly}
F4	Comment
F5	{1, No}, {2, Yes}
F6	{1, No}, {2, Yes}
F7	{1, No}, {2, Yes}
F7a	Comment
F8.1	{1, Strongly disagree}, {2, Rather disagree}, {3, Partly / partly}, {4, Rather agree}, {5, Strongly agree}
F8.2	{1, Strongly disagree}, {2, Rather disagree}, {3, Partly / partly}, {4, Rather agree}, {5, Strongly agree}
F9	{1, Yes}, {2, I'm not sure.}, {3, No}
F10	{1, Yes}, {2, I'm not sure.}, {3, No}
F11	{1, Very unsatisfied}, {2, Rather unsatisfied}, {3, Partly / partly}, {4, Rather satisfied}, {5, Very satisfied}
F12a	Comment
F12b	Comment
F12aa	{1, Very irrelevant}, {2, Rather irrelevant}, {3, Partly / partly}, {4, Rather relevant}, {5, Very relevant}
F12bb	{1, Very irrelevant}, {2, Rather irrelevant}, {3, Partly / partly}, {4, Rather relevant}, {5, Very relevant}
F13a	{1, Strongly disagree}, {2, Rather disagree}, {3, Partly / partly}, {4, Rather agree}, {5, Strongly agree}
F13b	{1, Strongly disagree}, {2, Rather disagree}, {3, Partly / partly}, {4, Rather agree}, {5, Strongly agree}
F14	Comment
F15a	{1, Not affected at all}, {2 Rather not ffected}, {3, Partly / partly}, {4, Rather affected}, {5, Very affected}
F15b	{1, Not affected at all}, {2 Rather not ffected}, {3, Partly / partly}, {4, Rather affected}, {5, Very affected}
F15c	{1, Not affected at all}, {2 Rather not ffected}, {3, Partly / partly}, {4, Rather affected}, {5, Very affected}
F15d	{1, Not affected at all}, {2 Rather not ffected}, {3, Partly / partly}, {4, Rather affected}, {5, Very affected}
F15e	{1, Not affected at all}, {2 Rather not ffected}, {3, Partly / partly}, {4, Rather affected}, {5, Very affected}
F15f	{1, Not affected at all}, {2 Rather not ffected}, {3, Partly / partly}, {4, Rather affected}, {5, Very affected}
F15g	{1, Not affected at all}, {2 Rather not ffected}, {3, Partly / partly}, {4, Rather affected}, {5, Very affected}
F16	{1, Not affected at all}, {2 Rather not ffected}, {3, Partly / partly}, {4, Rather affected}, {5, Very affected}
F17a	Comment
F17b	Comment
S1	Comment
S2	{1, Female}, {2, Male}, {3, Non-binary}
S3	{1, Single}, {2, Married}, {3, Registered partnership}, {4, Divorced}, {5, Registered partnership nullified}, {6, Widowed}, {7, Registered partner deceased}
S4	Comment
S5	Comment
S6	{1, I prefer not to disclose}, {2, No school-leaving qualification}, {3, Still in school}, {4, Lower secondary education}, {5, Polytechnic secondary education}, {6, Secondary education}, {7, University of applied sciences entrance qualification} {8, General university entrance qualification}, {9, Apprenticeship completed}, {10, Master /Technician}, {11, Bachelor's degree (university of applied sciences)}, {12, Master's degree (university of applied sciences)}, {13, Bachelor's degree (university)}, {14, Master's degree (university)}, {15, Doctoral degree}
S7	{1, I prefer not to disclose}, {2, <1,000 €}, {3, 1,000 € - 1,999 €}, {4, 2,000 € - 2,999 €}, {5, 3,000 € - 3,999 €}, {6, 4,000 € - 4,999 €}, {7, 5,000 € - 5,999 €}, {8, 6,000 € - 6,999 €}, {9, ≥7,000 €}
S8	{1, I prefer not to disclose}, {2, No confession}, {3, Christianity, Catholic}, {4, Christianity, Protestant}, {5, Islam}, {6, Judaism}, {7, Hinduism}, {8, Buddhism}, {9, Others, that is …}
S8a	Comment
S9	{1, < 2 years}, {2, 2 years - <4 years}, {3, 4 years - <6 years}, {4, 6 years - <8 years}, {5, 8 years - <10 years}, {6, 10 years - <12 years}, {7, 12 years - <14 years}, {8, 14 years - <16 years}, {9, 16 years - <18 years}, {10, 18 years - <20 years}, {11, ≥20 years}
S10	{1, Not at all}, {2, Once}, {3, Twice}, {4, 3 times}, {5, 4 times}, {6, 5 times}, {7, >5 times}
S11	{1, 1}, {2, 2}, {3, 3}, {4, 4}, {5, 5}, {6, >5}

Survey II

Participant no	Building	F0	F1	F2	F3	F4	F5	F6	F7	F7a
2	B	0	5	1	5		2	2	2	
3	B	0	5	1	4		2	2	2	Paper bag
14	B	0	5	3	5		2	2	2	Paper bag
1	B	1	4	2	4	Unaware of the difference between bio-waste and residual waste	2	2	1	Paper shopping bag
12	C	0	3	1	3		2	2	2	Paper bag
13	C	0	5	4	5		2	2	2	Paper bag
15	C	0	5	4	5	Disgusted by waste bins (especially in summer)	2	2	2	Plastic bag (emptied and disposed separately)
8	C	1	5	3	5	Packaging, Multilayer materials	2	2	1	
9	C	1	4	3	4		2	2	1	
10	C	1	4	2	4		2	2	2	Plastic bag (emptied and disposed separately)
7	A	0	4	1	5		2	2	2	Paper bag
16	A	0	4	3	5		2	2	2	Plastic bag
4	A	1	5	2	5		2	2	1	Paper / cardboard at the bottom
5	A	1	5	3	4		2	2	2	Plastic bag (emptied and disposed separately)
6	A	1	5	5	4		2	2	2	Plastic bag (emptied and disposed separately)
11	A	1	5	3	5		2	2	1	

	Building	F0	F1	F2	F3	F4	F5	F6	F7	F7a
Mean	A	0.67	4.67	2.83	4.67	-	2.00	2.00	1.67	-
Standard deviation	A	0.52	0.52	1.33	0.52	-	0.00	0.00	0.52	-
Mean	B	0.25	4.75	1.75	4.50	-	2.00	2.00	1.75	-
Standard deviation	B	0.50	0.50	0.96	0.58	-	0.00	0.00	0.50	-
Mean	C	0.50	4.33	2.83	4.33	-	2.00	2.00	1.67	-
Standard deviation	C	0.55	0.82	1.17	0.82	-	0.00	0.00	0.52	-
Mean	Overall	0.50	4.56	2.56	4.50	-	2.00	2.00	1.69	-
Standard deviation	Overall	0.52	0.63	1.21	0.63	-	0.00	0.00	0.48	-

Survey II

Participant no	Building	F8.1	F8.2	F9	F10	F11	F12a
2	B	5	4	1	2	10	
3	B	1	1	3	2	10	
14	B	1	1	1	2	10	
1	B	1	1	2	2	8	
12	C	3	2	2	2	6	
13	C	1	1	1	1	9	
15	C	1	1	1	2	7	Bins should be emptied more often in the summer time
8	C	1	1	1	2	8	
9	C	2	1	2	2	10	
10	C	1	1	1	1	9	
7	A	1	1	3	2	7	Waste collection works fine
16	A	1	1	1	2	9	
4	A	1	1	1	2	8	Sometimes bins are not put back in place after being emptied
5	A	1	1	1	1	9	
6	A	1	1	1	1	8	Sometimes bins are not put back in place after being emptied
11	A	1	1	1	1	8	Wishes for more decentral collection points; Regular collection of bulky waste
Mean	A	1.00	1.00	1.33	1.50	8.17	-
Standard deviation	A	0.00	0.00	0.82	0.55	0.75	-
Mean	B	2.00	1.75	1.75	2.00	9.50	-
Standard deviation	B	2.00	1.50	0.96	0.00	1.00	-
Mean	C	1.50	1.17	1.33	1.67	8.17	-
Standard deviation	C	0.84	0.41	0.52	0.52	1.47	-
Mean	Overall	1.44	1.25	1.44	1.69	8.50	-
Standard deviation	Overall	1.09	0.77	0.73	0.48	1.21	-

Survey II

Participant no	Building	F12b	F12aa	F12bb	F13a	F13b
2	B	Do their job, everything works well	5	5	5	5
3	B	Never had any problems, waste collection on time, tidy up	4	5	3	3
14	B	Tidiness	5	5	5	5
1	B	Waste collection works well, do a good job	3	3	3	3
12	C	Waste collection works well, take care of a tidy city	4	4	5	5
13	C	Recycling centers are well-operated, friendly, thorough, no complications	5	5	5	4
15	C	Enough waste bin capacity, new bins were provided	5	5	4	4
8	C	Everything works well	5	5	4	4
9	C	Lots of decentral collection points in the vicinity (textiles, glass), bins almost never at full capacity	5	5	4	3
10	C	Can always get a hold of them, good service	4	4	4	3
7	A		3	3	4	3
16	A	Pick up anything, put bins back in place	5	5	4	3
4	A	Regular collection, variances in collection dates are anounced via newspaper	5	5	5	4
5	A	Very good service and consulting, well-operated recycling center, waste collection on time	3	4	3	4
6	A	Do a good job	5	5	3	3
11	A	Easy to get a hold of in case of questions, working system	5	5	5	5
Mean	A	-	4.33	4.50	4.00	3.67
Standard deviation	A	-	1.03	0.84	0.89	0.82
Mean	B	-	4.25	4.50	4.00	4.00
Standard deviation	B	-	0.96	1.00	1.15	1.15
Mean	C	-	4.67	4.67	4.33	3.83
Standard deviation	C	-	0.52	0.52	0.52	0.75
Mean	Overall	-	4.44	4.56	4.13	3.81
Standard deviation	Overall	-	0.81	0.73	0.81	0.83

Survey II

Participant no	Building	F14	F15a	F15b	F15c	F15d
2	B		5.00	5.00	4.00	5.00
3	B	Climate friendly alternatives need to be real alternatives; trains often delayed	1.00	1.00	4.00	5.00
14	B		5.00	5.00	5.00	5.00
1	B	Daily implementation, do not travel by plane	4.00	1.00	4.00	4.00
12	C		3.00	1.00	1.00	3.00
13	C	Lack of will power	4.00	4.00	4.00	5.00
15	C	Intransparency of what is the more sustainable alternative, better insulation, limit own consumption, often means making sacrifices				
8	C	Means sacrifices	3.00	4.00	4.00	4.00
9	C	No sufficient alternatives	2.00	3.00	1.00	1.00
10	C		4.00	4.00	5.00	2.00
7	A	Not interested	1.00	2.00	3.00	3.00
16	A	Job (flight attendant)	4.00	1.00	4.00	3.00
4	A	Very aware of the room temperature, could probably save more water	4.00	4.00	4.00	4.00
5	A	Have two children, infrastructure	3.00	4.00	5.00	2.00
6	A	Would have to get rid of the car, infrastructure would have to be improved	3.00	4.00	4.00	3.00
11	A	Financial aspects	2.00	2.00	2.00	2.00
Mean	A	-	2.83	2.83	3.67	2.83
Standard deviation	A	-	1.17	1.33	1.03	0.75
Mean	B	-	3.75	3.00	4.25	4.75
Standard deviation	B	-	1.89	2.31	0.50	0.50
Mean	C	-	3.20	3.20	3.00	3.00
Standard deviation	C	-	0.84	1.30	1.87	1.58
Mean	Overall	-	3.20	3.00	3.60	3.40
Standard deviation	Overall	-	1.26	1.51	1.30	1.30

Survey II

Participant no	Building	F15e	F15f	F15g	F16	F17a
2	B	5.00	5.00	5.00	5.00	
3	B	1.00	4.00	4.00	3.00	Mindful use of collection bags, uses paper bags, pays more attention to waste separation
14	B	2.00	3.00	3.00	5.00	Pay more attention to waste separation (did not care before), inform themselves if unsure about proper disposal
1	B	1.00	3.00	1.00	4.00	Used to dispose any waste via bio-waste bins, now pay attention to waste separation
12	C	3.00	1.00	1.00	3.00	
13	C	4.00	2.00	1.00	4.00	Informed themselves about multimaterials, more aware of the importance of waste separation
15	C					
8	C	3.00	4.00	4.00	4.00	
9	C	1.00	2.00	2.00	3.00	Risen awareness, informed themselves about proper waste separation
10	C	1.00	3.00	2.00	4.00	Pay more attention to waste separation, reconsidered their own waste separation behaviour
7	A	2.00	3.00	1.00	2.00	
16	A	1.00	3.00	3.00	3.00	
4	A	3.00	2.00	2.00	4.00	
5	A	1.00	4.00	2.00	2.00	
6	A	1.00	5.00	5.00	3.00	Do not dispose of textiles via waste bins; apart from this, nothing changed
11	A	1.00	4.00	1.00	1.00	
Mean	A	1.50	3.50	2.33	2.50	-
Standard deviation	A	0.84	1.05	1.51	1.05	-
Mean	B	2.25	3.75	3.25	4.25	-
Standard deviation	B	1.89	0.96	1.71	0.96	-
Mean	C	2.40	2.40	2.00	3.60	-
Standard deviation	C	1.34	1.14	1.22	0.55	-
Mean	Overall	2.00	3.20	2.47	3.33	-
Standard deviation	Overall	1.31	1.15	1.46	1.11	-

Survey II

Participant no	Building	F17b
2	B	
3	B	Shopping behaviour has changed, use different collection bags than before, try to avoid plastic packaging and instead buy more items with paper packaging, risen awareness
14	B	
1	B	
12	C	
13	C	Impact on shopping behaviour, consider prospective waste disposal already while shopping
15	C	
8	C	
9	C	
10	C	
7	A	
16	A	
4	A	
5	A	
6	A	Meanwhile think that it is a pity that there not more plastic-free alternative products, risen awareness
11	A	
Mean	A	-
Standard deviation	A	-
Mean	B	-
Standard deviation	B	-
Mean	C	-
Standard deviation	C	-
Mean	Overall	-
Standard deviation	Overall	-

Survey II

Participant no	Building	S1	S2	S3	S4	S5	S6	S7	S8	S8a	S9	S10	S11
2	B	30	2	1	2	0	9	2	5		1	2	1
3	B	24	1	1	2	0	6	3	4		3	1	3
14	B	30	1	1	3	1	4	2	2		3	3	2
1	B	26	1	1	3	0	8	4	9	Paganism	5	7	6
12	C	16	1	1	3	2	8	1	3		1	1	2
13	C	29	2	1	2	0	14	7	4		11	2	3
15	C	24	2	2	2	0	8	4	4		11	2	2
8	C	57	1	2	2	0	6	1	2		11	1	2
9	C	28	2	1	2	0	14	7	2		3	6	3
10	C	32	2	2	2	0	9	4	4		11	3	1
7	A	30	2	1	2	0	1	1	2		11	1	1
16	A	55	2		2	0	13	6	2		2	2	6
4	A	82	1	2	2	0	14	1	3		9	1	2
5	A	45	2	2	4	2	9	6	4		11	4	3
6	A	75	1	1	1	0	14	1	2		11	4	3
11	A	60	1	4	2	1	8	3	4		11	5	6
Mean	A	57.83	1.50	2.00	2.17	0.50	9.83	3.00	2.83	-	9.17	2.83	3.50
Standard deviation	A	19.14	0.55	1.22	0.98	0.84	5.04	2.45	0.98	-	3.60	1.72	2.07
Mean	B	27.50	1.25	1.00	2.50	0.25	6.75	2.75	5.00	-	3.00	3.25	3.00
Standard deviation	B	3.00	0.50	0.00	0.58	0.50	2.22	0.96	2.94	-	1.63	2.63	2.16
Mean	C	31.00	1.67	1.50	2.17	0.33	9.83	4.00	3.17	-	8.00	2.50	2.17
Standard deviation	C	13.89	0.52	0.55	0.41	0.82	3.37	2.68	0.98	-	4.69	1.87	0.75
Mean	Overall	40.19	1.50	1.53	2.25	0.38	9.06	3.31	3.50	-	7.19	2.81	2.88
Standard deviation	Overall	19.73	0.52	0.83	0.68	0.72	3.89	2.21	1.79	-	4.32	1.91	1.71

Collection intervals before regular municipal waste collection as well as before waste characterization campaigns

Building	Bio-waste		Residual waste	
	Regular collection interval	Collection interval before characterization campaigns	Regular collection interval	Collection interval before characterization campaigns
	[d]			
A	14	13	7	6
B	14	9	14	6
C	14	13	7	7

Pictures of the measures implemented

Left side: Postal letter, multilingual information leaflet, and pre-sorting equipment (bins and paper collection bags).
Right side: Posters hung up in the hallways.

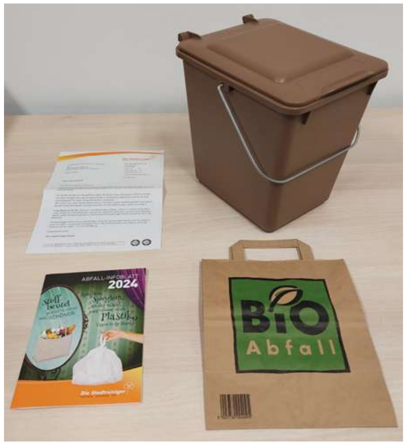

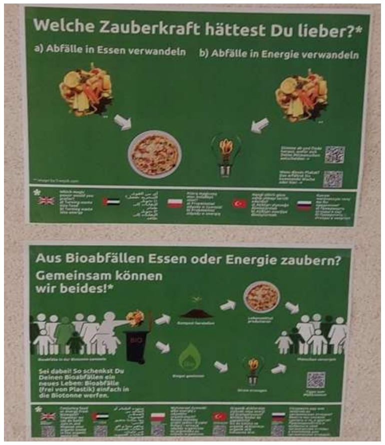

Location of investigated buildings in Kassel

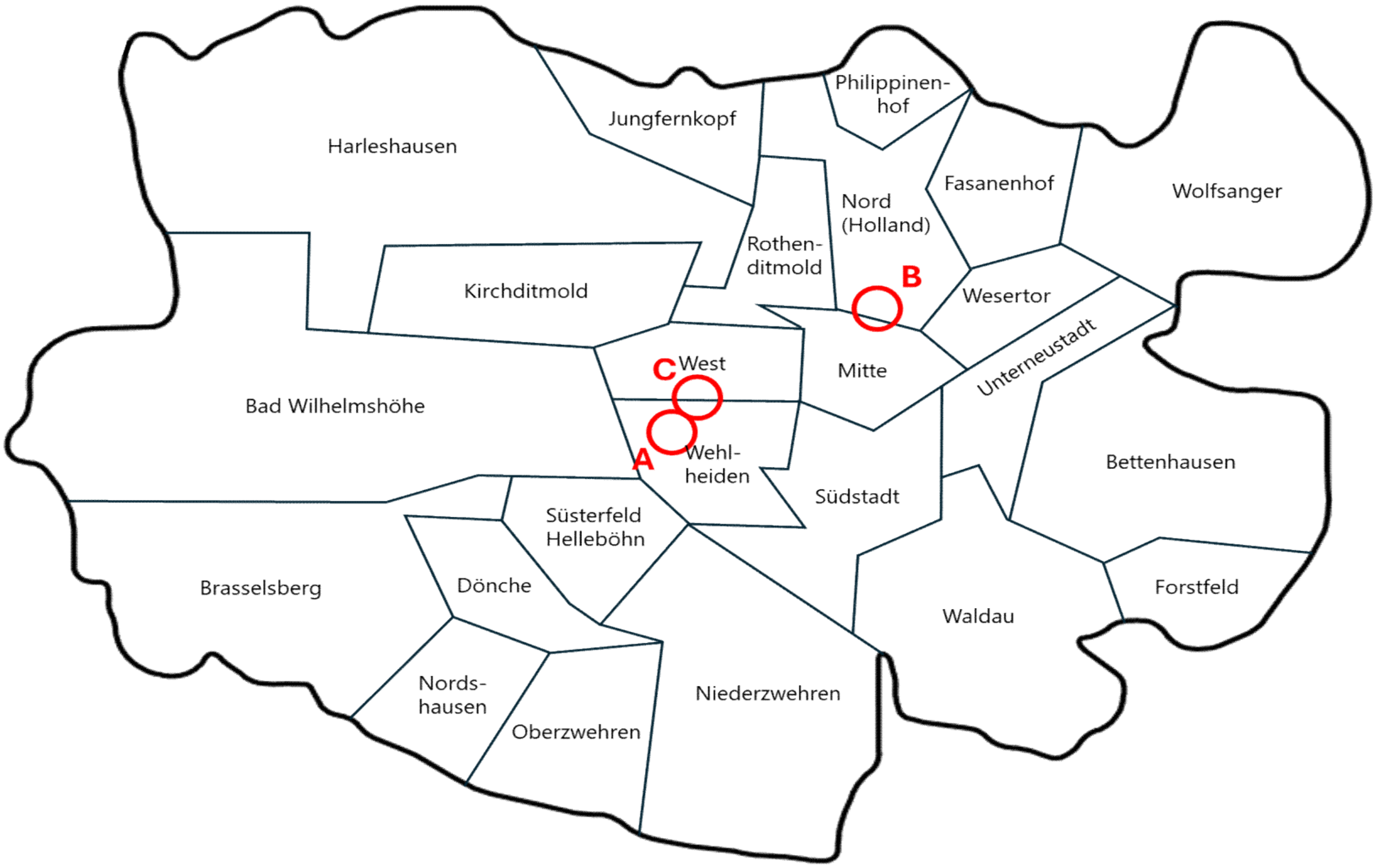

Supplement: sj-pdf-1-wmr-10.1177_0734242X261419233 – Supplemental material for Observation versus self-perception: Effectiveness of measures to increase separate bio-waste collection from multi-storey residential buildings [file sj-pdf-1-wmr-10.1177_0734242X261419233.pdf]
